# Data Anonymization for Pervasive Health Care: Systematic Literature Mapping Study

**DOI:** 10.2196/29871

**Published:** 2021-10-15

**Authors:** Zheming Zuo, Matthew Watson, David Budgen, Robert Hall, Chris Kennelly, Noura Al Moubayed

**Affiliations:** 1 Department of Computer Science Durham University Durham United Kingdom; 2 Cievert Ltd Newcastle upon Tyne United Kingdom

**Keywords:** healthcare, privacy-preserving, GDPR, DPA 2018, EHR, SLM, data science, anonymization, reidentification risk, usability

## Abstract

**Background:**

Data science offers an unparalleled opportunity to identify new insights into many aspects of human life with recent advances in health care. Using data science in digital health raises significant challenges regarding data privacy, transparency, and trustworthiness. Recent regulations enforce the need for a clear legal basis for collecting, processing, and sharing data, for example, the European Union’s General Data Protection Regulation (2016) and the United Kingdom’s Data Protection Act (2018). For health care providers, legal use of the electronic health record (EHR) is permitted only in clinical care cases. Any other use of the data requires thoughtful considerations of the legal context and direct patient consent. Identifiable personal and sensitive information must be sufficiently anonymized. Raw data are commonly anonymized to be used for research purposes, with risk assessment for reidentification and utility. Although health care organizations have internal policies defined for information governance, there is a significant lack of practical tools and intuitive guidance about the use of data for research and modeling. Off-the-shelf data anonymization tools are developed frequently, but privacy-related functionalities are often incomparable with regard to use in different problem domains. In addition, tools to support measuring the risk of the anonymized data with regard to reidentification against the usefulness of the data exist, but there are question marks over their efficacy.

**Objective:**

In this systematic literature mapping study, we aim to alleviate the aforementioned issues by reviewing the landscape of data anonymization for digital health care.

**Methods:**

We used Google Scholar, Web of Science, Elsevier Scopus, and PubMed to retrieve academic studies published in English up to June 2020. Noteworthy gray literature was also used to initialize the search. We focused on review questions covering 5 bottom-up aspects: basic anonymization operations, privacy models, reidentification risk and usability metrics, off-the-shelf anonymization tools, and the lawful basis for EHR data anonymization.

**Results:**

We identified 239 eligible studies, of which 60 were chosen for general background information; 16 were selected for 7 basic anonymization operations; 104 covered 72 conventional and machine learning–based privacy models; four and 19 papers included seven and 15 metrics, respectively, for measuring the reidentification risk and degree of usability; and 36 explored 20 data anonymization software tools. In addition, we also evaluated the practical feasibility of performing anonymization on EHR data with reference to their usability in medical decision-making. Furthermore, we summarized the lawful basis for delivering guidance on practical EHR data anonymization.

**Conclusions:**

This systematic literature mapping study indicates that anonymization of EHR data is theoretically achievable; yet, it requires more research efforts in practical implementations to balance privacy preservation and usability to ensure more reliable health care applications.

## Introduction

### Background

Digital health [1] encompasses several distinct domains, including but not limited to automatic visual diagnostic systems [2], medical image segmentation [3], continuous patient monitoring [4], clinical data–driven decision support systems [5-7], connected biometric sensors [8,9], and expert-knowledge–based consultations [10,11] using personal electronic health records (EHRs) [12-14]. Of late, pervasive health care has become the central topic, attracting intensive attention and interest from academia [2-4], industry [5,10,11], and the general health care sector [13-15]. Developments achieved in the industry [5] and the health care sector [12-14,16] reveal the huge potential of data science in health care because of the common availability of medical patient data for secondary use (secondary use, also dubbed as reuse, of health care data refers to the use of data for a different purpose than the one for which the data were originally collected). However, such potential could be hindered by legitimate concerns over privacy [17].

The United Kingdom’s Human Rights Act 1998 defines privacy as “everyone has the right to respect for [their] private and family life, [their] home and [their] correspondence” in Article 8 [18]. However, it is difficult to explicitly define true privacy because of the discrepancies among target problems, for example, human-action recognition from videos [19], camera-pose estimation from images [20], and next-word prediction from articles [21]. In general, privacy can be treated as any personally identifiable information [22,23]. In the context of digital health care, the secondary use of patients’ clinical data requires both the data controller (responsible for determining the purpose for which, and the means by which, health care data are processed) and data processor (responsible for processing health care data on behalf of the data controller) to comply with the lawful basis and gain direct consent from the data owner [24]. Recently, privacy invasion became an increasing concern in digital health care [25-28]. In 2014, the UK charity Samaritans (ie, data processor) released the app Radar [29] to identify potential distress and suicidality using the words and phrases of approximately 2 million Twitter (ie, data controller) users (ie, data owners). This app raised severe concerns among Twitter users, including those with a history of mental health issues, and thus it was pulled within weeks [26]. In 2015, the Royal Free London National Health Service (NHS) Foundation Trust (ie, data controller) shared 1.6 million complete and identifiable medical records of patients (ie, data owners) with DeepMind Technologies (Alphabet Inc; ie, data processor) to support further testing of the app Stream in assisting the detection of acute kidney injury [30]. This collaboration came under fire [27] for the inappropriate sharing of confidential patient data [24,31] and failure to comply with the United Kingdom’s Data Protection Act (DPA), as was ruled [32] by the Information Commissioner’s Office (ICO), which cited missing patient consent as well as lack of detailed purpose of use, research ethics approval, and the necessary process transparency [25]. Thus, a prerequisite for secondary use of clinical patient data is to guarantee patient privacy through data anonymization [33]. This is supported by legislation established in different countries that states that secondary use of clinical patient data is permitted if, and only if, the exchanged information is sufficiently anonymized in advance to prevent any possible future association with the data owners (ie, patients) [28,34]. For instance, researchers from academia pointed out the importance of patient-specific health data, which became the impetus for updating the United States’ Health Information Portability and Accountability Act (HIPAA) in 2003 [35,36].

On January 30, 2020, a declaration [37] by the World Health Organization named the COVID-19 [38] outbreak a Public Health Emergency of International Concern. At present (as of April 18, 2021), there are a total of 140,835,884 and 4,385,938 confirmed cases and 3,013,111 and 150,419 deaths, respectively, throughout the world [39] and the United Kingdom [40]. As COVID-19 spread to every inhabitable continent within weeks [41], data science research relating to digital health care through large-scale data collection [42,43] and crowdsourcing [44,45] has been highly recommended to curb the ongoing pandemic, including virus tracing [46,47] and contact tracing [48,49]. Public concern with respect to privacy has significantly increased amid the COVID-19 pandemic [50,51]. For instance, mobile apps have been adopted to make contact tracing and notification instantaneous upon case confirmation [52,53], for example, the latest NHS COVID-19 app [54]. This is typically achieved by storing a temporary record of proximity events among individuals and thus immediately alerting users of recent close contact with diagnosed cases and prompting them to self-isolate. These apps have been placed under public scrutiny over issues of data protection and privacy [48].

**Figure 1:**
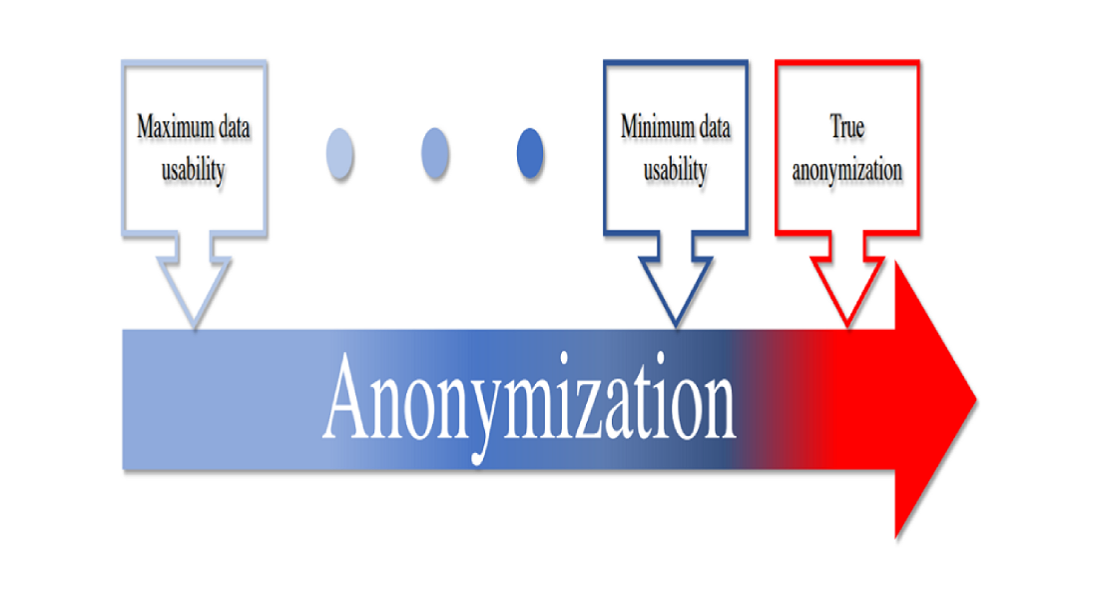
The curse of anonymization. Blue hue indicates an increase in data anonymity, which, in turn, reveals the decrease in usability of the anonymized data, very likely reaching minimum usability before reaching full anonymization (red hue).

Currently, the lack of more intuitive guidance and a deeper understanding of how to feasibly anonymize personally identifiable information in EHRs (it should be noted that data from wearables, smart home sensors, pictures, videos, and audio files, as well as the combination of EHR and social media data, are out of the scope of this study) while ensuring an acceptable approach for both patients and the public leave the data controller and data processor susceptible to breaches of privacy. Although several diligent survey papers [55-58] have been published to ensure privacy protection and suppress disclosure risk in data anonymization, sensitive information still cannot be thoroughly anonymized by reducing the risk of reidentification while still retaining the usefulness of the anonymized data—*the curse of anonymization* (Figure 1). Concretely, the gaps in the existing survey studies are four-fold: (1) there does not exist a single data anonymization survey that considers lawful aspects such as the European Union’s General Data Protection Regulation (GDPR) as well as the DPA, ICO, and health care provider regulations; (2) most existing survey studies do not focus on digital health care; (3) the existing privacy models are usually incomparable (particularly for the values of parameters) and have been proposed for different problem domains; and (4) the most recent trends of privacy model–based and machine learning–based data anonymization tools have not been summarized with adequate discussions in terms of their advantages and disadvantages. Motivated by these observations, we aim to deliver a clear picture of the landscape of lawful data anonymization while mitigating its curse in pervasive health care.

### A Brief Overview of the Problem Domain

#### Private Data and Their Categorization

In line with the updated scope of the GDPR and its associated Article 9 [59,60], private (ie, personal) data are defined as any direct or indirect information related to an identified or identifiable natural person. In general, based on the definition and categorization presented in chapter 10 of *Guide to the De-Identification of Personal Health Information* by El Emam [61], there are 5 types of data: relational data, transactional data, sequential data, trajectory data, and graph data. In addition, inspired by the survey study by Zigomitros et al [62], we also included image data because an EHR is essentially a 2D data matrix and thus could be viewed as a 2D image and anonymized using statistical and computer vision techniques.

Relational data [62] are the most common type of data. This category usually contains a fixed number of variables (ie, columns) and data records (ie, rows). Each data record usually pertains to a single patient, with that patient appearing only once in the data set. Typical relational data in health care can include clinical data in a disease or population registry. Transactional data [63] have a variable number of columns for each record. For instance, a data set of follow-up appointments from a hospital may consist of a set of prescription drugs that were prescribed to patients, and different patients may have a different number of transactions (ie, appointments) and prescribed drugs in each transaction. Sequential data [64] are similar to transactional data, but there is an order to the items in each record. For instance, a data set containing *Brachytherapy planning time* would be considered sequential data because some items appear before others. Sequential data can also be termed relational-transactional data. Trajectory data [65] combine sequential data with location information. For instance, data on the movement of patients would have location and timestamp information. Trajectory data can also be termed geolocational data. Graph data [66] encapsulate the relationships among objects using techniques from graph theory. For instance, data showing telephone calling, emailing, or instant messaging patterns between patients and general practitioners (GPs) could be represented as a graph, with patients and GPs being represented as nodes and a call between a given patient and their GP represented as an edge between their respective nodes. Graph data are also commonly used in social media [67]. Image data, as tabular medical records (ie, EHRs), can be treated as a grayscale image in 2D space. It should be noted that, in this study, the term image data does not refer to medical images such as computed tomography scans.

#### Types of Identifiers

How the attributes are handled during the anonymization process depends on their categorization [61]. All attributes contained in a table X are usually grouped into 4 types: direct identifying attributes *I*, indirect identifying attributes (ie, quasi-identifiers [QIs]) *Q*, sensitive attributes *S*, and other attributes *O* [61]. Refer to Multimedia Appendix 1 for the mathematical symbols and definitions used throughout this study.

Direct identifiers *I*, which are also termed direct identifying attributes, provide explicit links to data subjects and can be used to directly identify patients [68]. In practice, one or more direct identifying attributes can be assigned to uniquely identify a patient, either by themselves or in conjunction with other information sources. Typical examples of the former case include NHS number, national insurance number, biometric residence permit number, and email address. Suppose there are 2 patients with the same full name within a single NHS foundation trust, the attribute *full name* cannot be a direct identifier by itself. However, a combination of *full name* and *living address* will be a direct identifier.

Indirect identifiers *Q*, or QIs, are identifiers that, when used with background knowledge of patients in the anonymized data set, can be used to reidentify a patient record with a high probability. Note that if someone, say, an adversary, does not have background knowledge of patients at hand, then this attribute cannot be deemed a QI. In addition, a common choice of QI also considers the analytical utility of the attribute. That is, a QI is usually useful for data analysis, whereas a direct identifier is not [61]. Typical QIs include gender, date of birth, postcode, and ethnic origin.

Sensitive attributes *S* are not useful with respect to the determination of the patient’s identity; yet, they contain sensitive health-related information about patients, such as clinical drug dosage. Other attributes *O* represent variables that are not considered sensitive and would be difficult for an adversary to use for reidentification.

**Figure 2:**
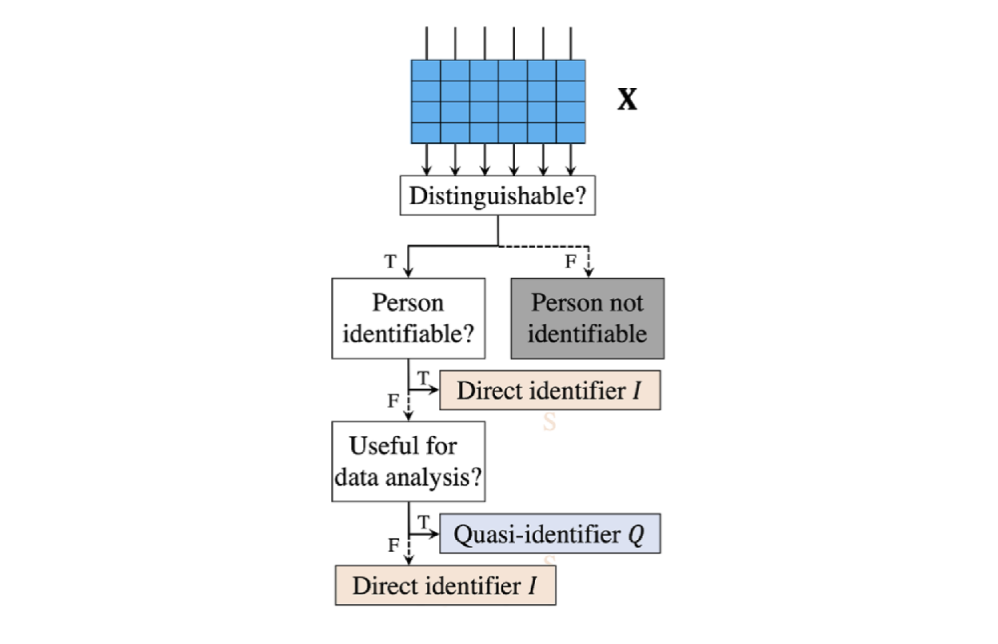
Logical flow of distinguishing direct identifiers I from quasi-identifiers Q. F: false; T: true.

Among the 4 categories of identifiers, it is particularly difficult to differentiate between direct identifiers *I* and QIs *Q* In general, there are 3 determination rules used for this purpose [61], which are depicted in Figure 2: (1) an attribute can be either *I* or *Q* if it can be known by an adversary as background knowledge; (2) an attribute must be treated as *Q* if it is useful for data analysis and as *I* otherwise; and (3) an attribute should be specified as *I* if it can uniquely identify an individual.

In Multimedia Appendix 2 [69,70], we summarize the features that are commonly listed as direct and indirect identifiers by health care bodies [71] that guide, inform, and legislate medical data release. All listed features may lead to personal information disclosure, and the list is by no means exhaustive. As more varied health care data are released and explored, more identifiers will be added to the lists of those featured in common data attack strategies, such as those in the studies by Hrynaszkiewicz et al [69] and Tucker et al [70], 18 HIPAA identifiers [72], and policies published by the NHS [73] and its foundation trusts, for example, Kernow [74] and Solent [75].

#### Data Anonymization Versus Data Pseudonymization

Given the definition in Recital 26 [76] of the most recent GDPR update, data anonymization (the term is common in Europe, whereas deidentification is more commonly used in North America) is a useful tool for sharing personal data while preserving privacy. Anonymization can be achieved by changing identifiers through removal, substitution, distortion, generalization, or aggregation. In contrast, data pseudonymization is a data management and deidentification procedure by which personally identifiable information fields within a data record are replaced by one or more artificial identifiers or pseudonyms.

It should be noted therefore that the relationship between data anonymization and pseudonymization techniques is characterized as follows:

- Anonymized data are not identifiable, whereas pseudonymized data are identifiable.
- Pseudonymized data remain personal based on Recital 26 of the GDPR and the conclusion [77] provided by the ICO.
- Solving the problem of data anonymization necessarily means solving pseudonymization.

Concretely, given an anonymization function *A* and raw data X, we have the anonymized data X’=*A(*X*)* such that there does not exist another function *R* that reidentifies the raw data X from the anonymized data X’, that is, *R*(X’)=*R*(*A*(X))=X. If such a function does exist, this is pseudonymization. The difference between these 2 operations can be generalized as follows: X→X’ for anonymization and X→X’.

In a real-world scenario, efficient data anonymization is challenging because it is usually problem dependent (ie, solutions vary across problem domains) and requires substantial domain expertise (eg, to specify the direct and indirect identifiers present in raw data) and effort (eg, user involvement in specifying the privacy model before the data anonymization process). Fundamentally, it is very challenging and nontrivial to define what *true anonymization* is or, equivalently, to determine whether the raw data have been adequately anonymized (as well as to agree upon the definition of *adequate anonymization*). In practice, as visualized in Figure 1, we observe that as the level of data anonymity increases, the usability of the anonymized data decreases and very likely reaches minimum usability before reaching full anonymization. This fact combined with the need for more accurate models in health care provides sufficient motivation for continued research into methods of data anonymization. For this study, we believe that how anonymization is defined is problem dependent. We reiterate that there is no clear-cut line between pseudonymization and anonymization because even anonymized data can practically have different reidentification risks [78,79] (depending on the type of anonymization performed).

### Aims of the Study

#### Objectives

To minimize bias and deliver up-to-date studies related to data anonymization for health care, we organized this survey in a systematic literature mapping (SLM) manner. In general, there are 2 main approaches to conduct literature reviews: systematic literature review (SLR) and SLM [80-82]. SLRs aim to identify, classify, and evaluate results to respond to a specific review question (RQ), whereas SLMs seek to investigate multiple RQs. In addition, SLRs synthesize evidence and consider the strength of such evidence [83], whereas an SLM provides an overview of a research area by reviewing the topics that have been covered in the literature [84]. Concretely, we combined high-quality systematic review studies—provided in the Cochrane Database of Systematic Reviews [85], Manchester; Centre for Reviews and Dissemination [86], York; and Health Technology Assessment [87], National Institute for Health Research—to explain this work explicitly and concisely with respect to the validity, applicability, and implication of the results.

Our overall objective is to alleviate the issues introduced toward the end of the previous section by reviewing the landscape of data anonymization for digital health care to benefit practitioners aiming to achieve appropriate trade-offs in leveraging the reidentification risk and usability of anonymized health care data. In other words, we evaluate the evidence regarding the effectiveness and practicality of data anonymization operations, models, and tools in secondary care from the perspective of data processors.

#### Defining RQs

The aims of the study are to evaluate the potential of preserving privacy using data anonymization techniques in secondary care. Concretely, we, as data processors, are highly motivated to investigate the best possible way of anonymizing real-world EHRs by leveraging the privacy and usability concerns visualized in Figure 1. Therefore, our RQs were defined as follows:

- RQ 1: Do best practices exist for the anonymization of realistic EHR data?
- RQ 2: What are the most frequently applied data anonymization operations, and how can these operations be applied?
- RQ 3: What are the existing conventional and machine learning–based privacy models for measuring the level of anonymity? Are they practically useful in handling real-world health care data? Are there any new trends?
- RQ 4: What metrics could be adopted to measure the reidentification risk and usability of the anonymized data?
- RQ 5: What are the off-the-shelf data anonymization tools based on conventional privacy models and machine learning?

The knowledge generated from this SLM, especially the answer to our driving question, RQ 1, will build on the study’s evidence on the future of the development of data anonymization toolkits for data processors such as the companies and organizations in which they are situated. The evidence gained may also contribute to our understanding of how data anonymization tools are implemented and their applicability to anonymizing real-world health care data. Finally, we intend to identify the major facilitators and barriers to data anonymization in secondary care in relation to reidentification risk and utility.

## Methods

### Research Design

#### Data Sources and Search Strategy

In keeping with our RQs, we built up our search strategy using *keywords and indexing terms* and *Boolean operators*; the former refers to the general terms used when searching, and the latter represents the restrictions on these terms. Example keywords and indexing terms used included domain-specific terms such as *healthcare*, *digital health*, *digital healthcare*, *health monitoring*, and *eHealth*; problem-specific terms such as *data anonymization*, *anonymizer*, *de-identification*, *privacy-preserving*, and *data protection*; data-specific terms such as *electronic medical records*, *electronic health records (EHR)*, *DICOM/CT images*, and *videos*; disease-specific terms such as *brain tumor*, *cervical cancer*, *breast cancer*, and *diabetes*; organization-specific terms such as *NHS*, *ICO*, *NIHR*, and *MRC*; and law-specific terms such as *DPA*, *GDPR*, and *HIPAA*. Example Boolean operators are *AND* and *OR*. Next, to avoid bias and ensure reliability, 2 researchers (ZZ and MW) used Google Scholar, Web of Science, Elsevier Scopus, and PubMed for searching academic studies up to June 2020; these services were used because they encompass a wide spectrum of databases such as IEEE Xplore, SpringerLink, ACM Digital Library, Elsevier Science Direct, arXiv, *The BMJ*, *Lancet*, and the *New England Journal of Medicine*. In addition, to maximize search coverage, we conducted forward and backward *snowball sampling* [88] (snowball sampling refers to using the reference list of a selected paper [backward snowballing] or the citations of a selected paper [forward snowballing]) on the selected studies. In particular, because gray literature is an important source of SLRs and SLMs [89] and they play a primary role in health care [90,91], gray literature was used to initialize our search in this study. Concretely, preprints from non–peer-reviewed electronic archives (eg, arXiv) or early-stage research were examined and distinguished in the follow-up study selection phase.

#### Inclusion and Exclusion Criteria

Articles were eligible for inclusion based on the criteria defined in Textbox 1. ZZ and MW assessed articles independently for inclusion eligibility. Inclusion is relatively straightforward in comparison with exclusion which can be more sweeping. Therefore, further clarification regarding some of the exclusion criteria is required. For instance, *without Experiment section* denotes that the article does not report on any evaluation of the ideas it contains using real-word clinical data sets. *Insights not suitable for EU/UK* indicates observations delivered by articles that treat personally identifiable data as a commercial commodity, as is the practice in, for example, the United States [92]. Preprints (tier 2 gray literature [93]) were carefully considered for selection in line with the inclusion and exclusion criteria summarized in Textbox 1. For duplicate articles (eg, a conference article that extended to a journal paper or a preprint paper accepted by either a conference or a journal), including those with a different title but essentially the same content, we only retained the publication with the highest quality to avoid double counting. To this end, we preferred to retain the article published by the journal with the highest impact factor. In the worst case, none of the duplicates would have been selected if they were all conference papers because this would have been a breach of research ethics.

**Inclusion and exclusion criteria for article selection.**

**Inclusion criteria**

- Related to anonymization or privacy-preserving techniques
- Related to privacy-preserving techniques in health care
- Presented privacy concerns in health care
- Proposed methods for privacy preservation in electronic health records
- Proposed methods for using private information, for example, biometric data
- Proposed methods partially related to protected health care

**Exclusion criteria**

- Written in language other than English
- Without *Abstract* or *Experiment* section
- About other health care issues, for example, clinical trials
- Insights not suitable for European Union or United Kingdom
- Out of our research scope
- Duplicate articles (case dependent)

#### Article Selection Phases

**Figure 3:**
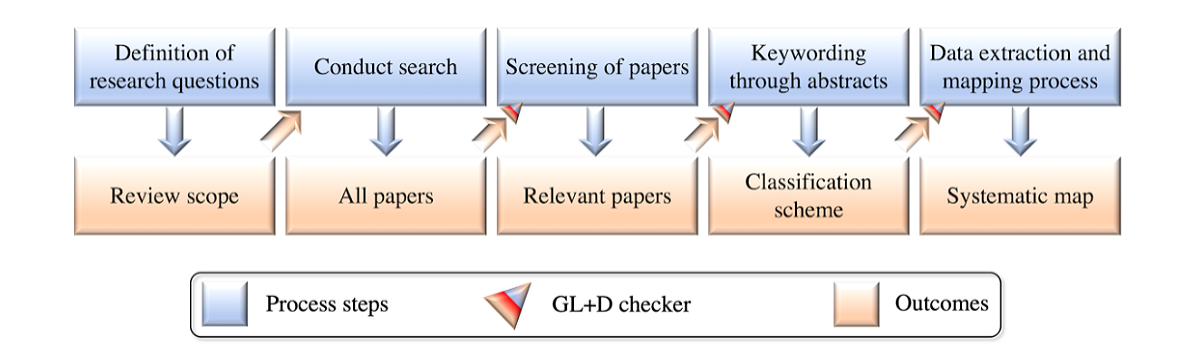
Systematic literature mapping process for articles. GL+D: gray literature and duplicates.

Article selection (Figure 3) consisted of 5 phases: (1) initially, we searched Google Scholar, Web of Science, Elsevier Scopus, and PubMed; (2) next, we applied the inclusion-exclusion criteria to the returned results from the initial search, including the qualifying preprints; (3) we then read the included articles and removed the irrelevant articles; (4) next, we conducted forward and backward snowball sampling on highly related articles; (5) finally, we double-checked the excluded articles and added relevant ones. In addition, we used the *GL+D Checker* mechanism shown in Figure 3, which refers to a combination of a *Gray Literature Checker* and a *Duplicates Checker*, each of which could also be used separately, depending on the situation.

#### Data Anonymization Toolkit Selection Phases

As mentioned at the beginning of this section, the phases involved in selecting data anonymization software tools are difficult because of the limited tools available in the existing studies. Thus, the initially included tools were selected from the qualified articles without considering whether their source code was publicly accessible, maintainable, and extensible. The only criterion was whether the tool could be downloaded and executed. Furthermore, to guarantee that the selection process was less biased, we decided that in each of the 2 (ie, privacy model–based and machine learning–based) categories of privacy-preserving software tools, the number of tools chosen from outside of the selected articles would be no more than 30% of the total.

### Conduct of the Study

#### Qualified Articles

**Figure 4:**
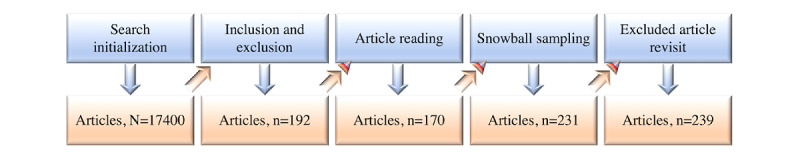
Number of selected articles during the study selection process.

**Figure 5:**
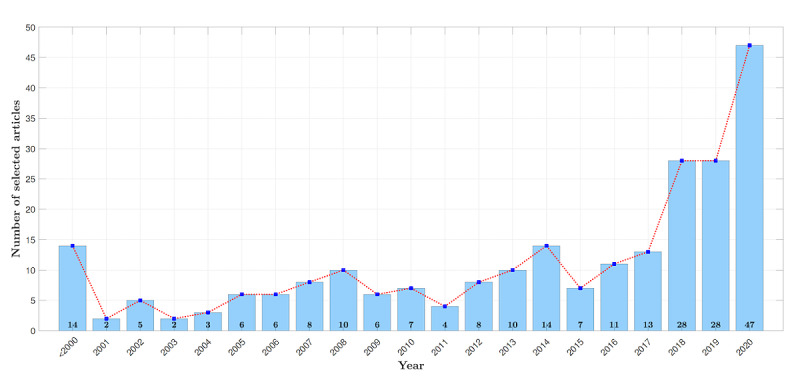
An overview of the 239 selected research articles grouped in chronological order.

In keeping with the five-phase article selection strategy described in the previous section, ZZ and MW independently selected articles for eligibility in phase 2. Articles were moved forward to the *Article reading* phase or excluded after a full agreement was reached. In addition, NAM served as an arbitrator for any unresolved disagreement. The selection process was conducted using 3 consecutive steps: (1) the title and abstract of each article were screened for relevance; (2) full article contents were reviewed for those without certainty for inclusion; and (3) forward and backward snowballing was applied to the remaining articles to maximize search coverage. The full reference list of the included articles and the related systematic review or mapping studies were also screened by hand for additional articles. There were a total of 13 preprints among the 192 selected articles (Figure 4) after phase 1. Before beginning phase 2, by applying the *Gray Literature Checker* mechanism, we observed that 4 of the 13 preprints had been successfully published in either peer-reviewed conferences [94-96] or journals [97]. Next, the *Duplicates Checker* was applied consecutively to remove their preprint versions. Using the same process in each phase, we accumulated a total of 239 articles to include in this SLM study, including 9 preprints. Details of the 239 selected research articles are grouped in categorical order and chronological order in Table 1 and Figure 5, respectively.

**Table 1:** An overview of the 239 selected research articles grouped in categorical order.

Category	Selected research articles, n (%)
Background knowledge	60 (25.1)
Data anonymization operations	16 (6.7)
Privacy models	104 (43.5)
Risk metrics	4 (1.7)
Utility metrics	19 (7.9)
Data anonymization tools	36 (15.1)

#### Qualified Software Tools

In accordance with the strategy of selecting qualified privacy-preserving software tools described in the previous section, there were 5 out of a total of 15 privacy model–based data anonymization tools that were not derived from the qualified (ie, selected) articles. Of these 5 tools, 3 (*Amnesia,* OpenAIRE; *Anonimatron,* realrolfje; and *Anonymizer,* Divante Ltd) were obtained by searching GitHub [98], and the remaining 2 (*OpenPseudonymiser,* Julia Hippisley-Cox and *NLM-Scrubber* from the US National Library of Medicine) were found through Google Search. Of the 5 machine learning–based tools, only one (*CrypTen*, Facebook Inc) was obtained from GitHub.

## Results

### Four Categories

To add structure to this SLM, we grouped the results of the reviewed articles into four categories: *Basic Data Anonymization Operations* (for RQ 2), *Level of Anonymity Guarantees and Evaluations* (for RQ 3), *Disclosure Risk Assessments* and *Usability Measurements* (for RQ 4), and *Existing Privacy Model*–*Based Data Anonymization Tools*, *Existing Machine Learning*–*Based Data Anonymization Tools*, and *Legal Framework Support* (for RQ 5). RQ 1, as the leading RQ, is answered in *Results Summary for RQs*.

### Basic Data Anonymization Operations

#### Perturbation

This technique is implemented by modifying the original data in a nonstatistically significant fashion. As described in the code of practice [99] provided by the ICO, the alteration of values within the data set should decrease the vulnerability of that data set to data linkage. The benefit of this method is that it anonymizes the raw data while guaranteeing that the statistical usefulness of the data remains unchanged. On this basis, the possible drawback of such a method is the accuracy of the anonymized data.

This technique can be achieved through, for instance, microaggregation [100], data swapping [101] (equivalent to permutation [102]), rank swapping [103]), postrandomization [104], adding noise [105], and resampling [106], all of which are described, with real-world health care examples to explain each operation, in Multimedia Appendix 3 [100,101,104-109]. For microaggregation, an observed value is replaced with the average value calculated over a small group of units. The units belonging to the same group are represented by the same value in the anonymized data. This operation can be applied independently to a single variable or to a set of variables with the original column or columns removed. For data swapping, the data records are altered through the switching of variable values across pairs of records in a fraction of the raw data. Equivalently, permutation rearranges the values (either randomly or systematically) and is useful where mapping to alternate configurations of alphanumeric values is problematic or redundant. To this end, the raw data can be efficiently perturbed by permuting the sensitive attribute and the value of a similar record. This operation not only guarantees the statistical significance of the anonymized data but also reduces the risk of the record-wise reidentification. For postrandomization, categorical variables are perturbed based on a prescribed probability mechanism such as a Markov matrix. For raw numerical data with low variance, adding noise, that is, adding a random value, is commonly adopted. Alternatively, resampling is also frequently used on raw numerical data by drawing repeated samples from the original data.

#### Generalization

Generalization [107] relies on an observable attribute having an underlying hierarchy. This is an example of such a typical hierarchy:

Full postcode → street → city or town → county (optional) → country

with a possible instance being as follows:

DH1 3LE → South Road → Durham → UK

and

DH → Durham → UK

Typically, generalization is used to reduce the specificity of the data and thereby the probability of information disclosure. Given the examples above, the degree of generalization is fully controlled by the granularity defined in the hierarchy.

#### Suppression

Suppression [110] refers to local suppression in data anonymization research. This is usually achieved by replacing the observed value of one or more variables with *missing* or *NA* or *-*. This method helps to address problems where rows would be dropped because of the difficulty of successfully applying perturbation or other generalization methods to guarantee their inclusion in the anonymized data set. By suppressing categorical values that render the rows identifiable, useful data from those rows will not be lost. This method can only be used when the raw data are varied enough that they prevent the suppressed value from being inferred.

#### Data Masking

Data masking [108] is a technique frequently used for creating a structurally similar yet inauthentic version of the raw data. This technique helps to protect the original sensitive data while providing a functional substitute and should be used in settings in which the original raw data are not required.

#### Differential Privacy

Differential privacy (DP) [109] aims to help organizations better understand the requirements of end users by maximizing the accuracy of search queries while minimizing the probability of identifying personal data information. This is achieved in practice by performing techniques such as data filtering, adaptive sampling, adding noise by fuzzifying certain features, and analyzing or blocking intrusive queries. Essentially, a DP algorithm updates values, leaving some intact while replacing others such that a potential attacker is unable to determine whether a value is fake or genuine. For details about practical DP and related techniques, please refer to section 1.4 of Multimedia Appendix 4 [57,66,111-165].

#### Homomorphic Encryption

Homomorphic encryption (HE) [166] is a technique that enables calculations to be performed on encrypted data directly, without the need to decrypt the data. The drawbacks of such a method are slow execution speeds. To the best of our knowledge, and in accordance with the definitions used in this paper, a technique that uses an encryption method cannot be treated as anonymization. The presence of the *key* makes the data theoretically reversible and therefore constitutes data pseudonymization. A well-known extension of HE is termed additive HE, which supports secure addition of numbers given only the encrypted data [167].

#### Compressive Privacy

Compressive privacy (CP) [168] is a technique that proposes to perform privatization by mapping the original data into space with a lower dimension. This is usually achieved by extracting the key features required for the machine learning model before sending the data to the cloud server. To this end, data owners (eg, NHS trusts and authorized companies) have control over privacy [169]. Alternatively, this technique could be performed before applying the chosen privacy models. Essentially, CP can be treated as a dimensionality reduction technique that also preserves privacy. Privacy models related to CP are presented in the following section.

### Level of Anonymity Guarantees and Evaluations

#### Measurement and Evaluation

##### Two Models

**Figure 6:**
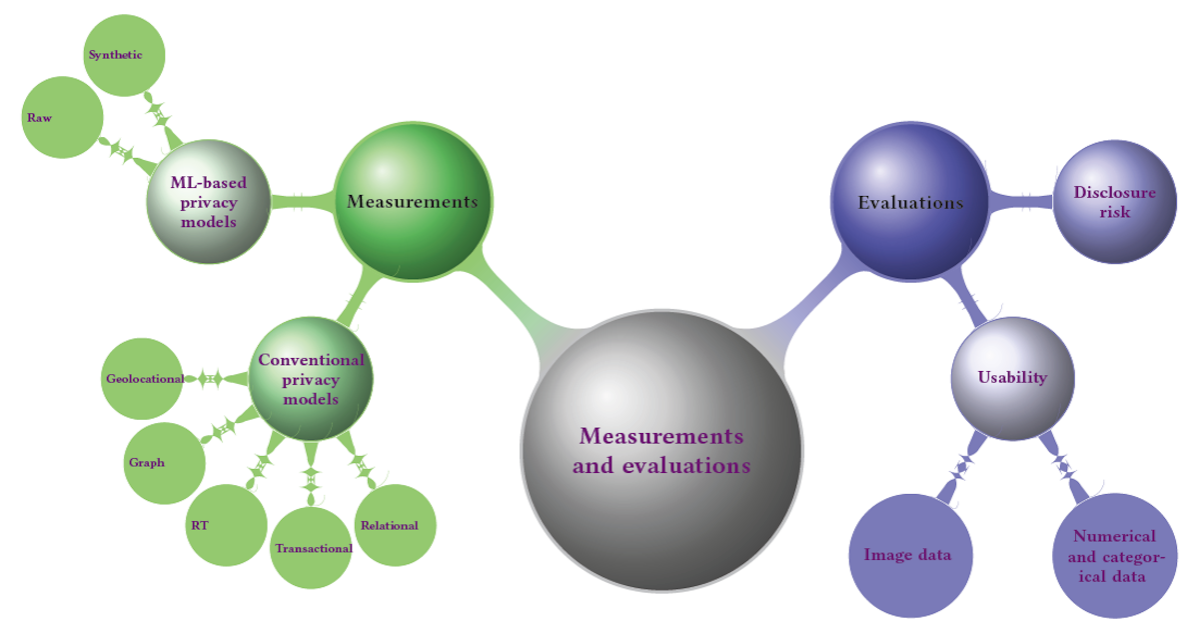
Categorizations of measurements and evaluations for achieving different levels of anonymity. ML: machine learning; RT: relational-transactional privacy model.

The objective of satisfying different levels of anonymity is usually achieved through 2 consecutive steps: measurement and evaluation. The former refers to the use of either conventional or machine learning–based privacy models to perform data anonymization, and the latter is the process of evaluating the reidentification risk and degree of usability of the anonymized data. The anonymization operations are usually adopted by conventional privacy models or machine-learning–based models. Figure 6 provides a way to quickly locate content of interest.

##### Conventional Privacy Models

The attributes contained in a table are usually divided into direct identifiers *I*, QIs *Q*, and sensitive identifiers *S*. The direct identifiers *I* are usually removed at the very beginning stage of data anonymization. Thus, a table X required to be anonymized is denoted as X(*S*, *Q*).

Given a class of records *G* in a table X, we want to create a single equivalent group *C* using a function *A* such that *C*=*A*(*G*) or *C’*=*A*(*C*). The monotonicity property of privacy models is defined for a single equivalent group *C* or class of records *G*. This property is required by several models for the purpose of refining the level of anonymization of *C*. This property is also useful for manipulating anonymized data by converting it into coarse-grained classes with equivalent classes (ie, a set of anonymized data records that share the same *Q*). This is a simple and computationally inexpensive solution. However, it would be inefficient, particularly in a case where the anonymized data are released to several organizations, each of which has a different minimum acceptable degree of anonymity. To this end, it is always a good practice to first perform the anonymization and then generate multiple coarser versions of the data, rather than performing separate anonymization for each organization [170].

**Figure 7:**
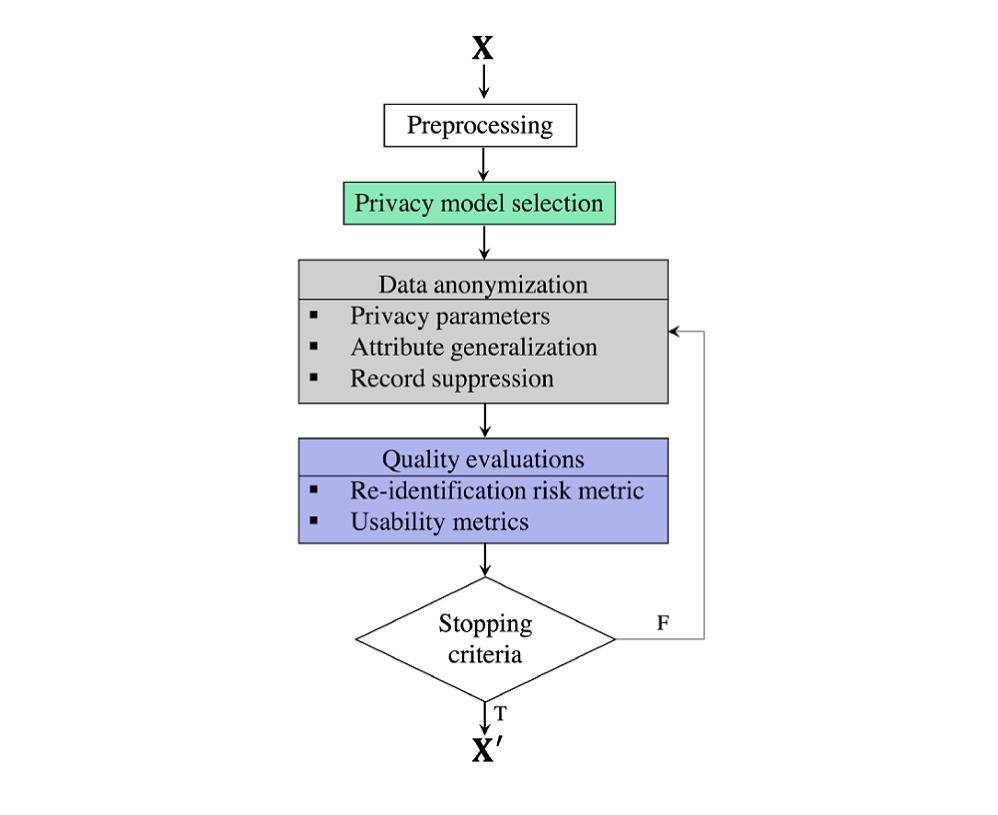
General pipeline for existing privacy model–based data anonymization tools. F: false; T: true.

During the process of data anonymization, interpretable and realistically feasible measurements (ie, privacy models [171]) should be considered to measure the level of anonymity of the anonymized data. The off-the-shelf privacy models (summarized as part of Figure 7) are usually independent of any data deanonymization attack and measure the privacy level using features of the anonymized data. One step further, 35 conventional privacy models were investigated to support data with the types grouped into 5 categories (Table 2).

**Table 2:** A summary of privacy models for relational electronic health record data with respect to parameter interval and degree of privacy of data.

Category	Privacy model	Section in Multimedia Appendix 4	Parameter interval	Privacy level	References
**Relational**					
	κ-anonymity	1.1	[1, |X|]	High	[111-117,172,173]
	(α, *k*)-anonymity	1.1.1	α ∈ [0, 1], *k* ∈ [0, +∞]	α: low, *k*: high	[114,174]
	*k*-map	1.1.2	[1, |X|]	Low	[112,175]
	*m*-invariance	1.1.3	[0, +∞]	High	[176]
	(*k*, *e*)-anonymity	1.1.4	[0, +∞]	High	[57,118,177]
	(*k*, *g*)-anonymity	1.1.5	*k* ∈ [0, +∞], *g* ∈ [0, 1]	High	[178,179]
	Multirelational *k*-anonymity	1.1.6	[0, +∞]	High	[180]
	Strict average risk	1.1.7	N/A^a^	Low	[181,182]
	*l*-diversity	1.2	[0, +∞]	High	[119]
	*l*^+^-diversity	1.2.1	*l* ∈ [0, +∞], *θ* ∈ [0, 1]	High	[120]
	*t*-closeness	1.3	[0, +∞]	Low	[121,122]
	Stochastic *t*-closeness	1.3.1	*t* ∈ [0, +∞], ε ∈ [0, +∞]	Low	[123]
	(*c*, *t*)-isolation	1.3.2	[0, +∞]	High	[124]
	β-Likeness and enhanced β-likeness	1.3.3	[0, +∞]	High	[125]
	Differential privacy	1.4	[0, +∞]	Low	[109]
	(*k*, ε)-anonymity	1.4.1	[0, +∞]	High	[126-131]
	(ε, δ)-anonymity	1.4.2	ε ∈ [0, +∞], δ ∈ [0, +∞]	ε: low, δ: low	[132-137]
	(ε, *m*)-anonymity	1.4.3	ε ∈ [0, 1], *m* ∈ [1, +∞]	ε: high, *m*: high	[118]
	Distributed differential privacy	1.4.4	[0, +∞]	Low	[138]
	Distributional differential privacy	1.4.5	ε ∈ [0, +∞], δ ∈ [0, +∞]	ε: low, δ: low	[139]
	*d*-χ-privacy	1.4.6	[0, +∞]	Low	[140]
	Joint differential privacy	1.4.7	ε ∈ [0, +∞], δ ∈ [0, +∞]	ε: low, δ: low	[183]
	(*X*, *Y*)-anonymity	1.5.1	[0, 1]	Low	[141]
	Normalized variance	1.5.2	[0, 1]	High	[142]
	δ-disclosure privacy	1.5.3	[0, +∞]	High	[143]
	(*d*,*y*)-privacy	1.5.4	[0, 1]	Low	[144,145]
	δ-presence	1.5.5	[0, 1]	Low	[57,146]
	Population and sample Uniqueness	1.5.6 or 1.5.7	N/A	N/A	[79,147-151]
	Profitability	1.5.8	N/A	N/A	[152]
Transactional	*k^m^*-anonymity	2	N/A	N/A	[153]
Relational-transactional	(*k*, *k^m^*)-anonymity	3	N/A	N/A	[154]
**Graph**					
	*k*-degree	4.1	N/A	N/A	[155-158]
	*k*^2^ degree	4.2	N/A	N/A	[156]
	*k*-automorphism	4.3	N/A	N/A	[157,159,160]
	(*k*, *l*)-anonymity	4.4	N/A	N/A	[66,161,162]
Geolocational	Historical *k*-anonymity	5	N/A	N/A	[163]

^a^N/A: not applicable.

##### Machine Learning–Based Privacy Models

##### Two Categories

In light of machine learning and its derived subset, deep learning, there has been an upsurge of interest in machine learning– or deep learning–based privacy models for anonymizing patient or general data; we explore these approaches in this section. We divided related machine learning–based privacy models into 2 categories in accordance with the type of data used: raw or synthetic. Of late, the use of synthetic data has become more popular because these generated data are both anonymous and realistic; therefore, consent from data owners is not required [184]. The data in this category can be generated using techniques such as generative adversarial networks (GANs) [185] and usually do not have the risk of reidentification; thus, research works concentrate on improving the utility of synthetic data.

##### Models for Raw Data

In the study by D’Acquisto and Naldi [186], conventional principal component analysis (PCA) was used to anonymize sensitive data sets to achieve anonymization-utility trade-offs, that is, maximize both the information loss and utility. Different from its use in reducing the dimension of the data, where the smallest principal components are removed, PCA was instead adopted to remove the largest principal components before data projection. To measure the usefulness of the data anonymized through PCA, several utility metrics were presented; these are discussed in detail in Multimedia Appendix 5 [117,172,186-213]. In the domain of data anonymization, the first work using PCA is termed as differentially private PCA [214]. This technique explores the trade-off between the privacy and utility of low-rank data representations by guaranteeing DP. The study by Dwork et al [215] suggested that noise be added directly to the covariance matrix before projection in PCA.

Many similar PCA techniques rely on results derived from random matrix theory [216-219]. To reduce the computational cost of the privacy model, additive HE was used for PCA with a single data user [217], where the rank of PCA with an unknown distribution could be adaptively estimated to achieve (𝜖, 𝛿)-DP [218]. More recently, the concept of collaborative learning (or shared machine learning) [94,97,220] became very popular in data anonymization. That is, the data collected from multiple parties are collectively used to improve the performance of model training while protecting individual data owners from any information disclosure. For instance, both HE and secret sharing were adopted in privacy-preserving PCA [219] for horizontally partitioned data, that is, data sets share the same feature space but different sample space. In that work, HE could be substituted with secure multiparty computation (SMPC) [221] for industrial use (more details are provided in *SMPC Frameworks* under *Results*).

Despite the great success achieved by PCA and its variants in data anonymization, traditional clustering algorithms have also been adopted to deal with the same problem; 𝑘-means [222], fuzzy c-means [223,224], Gaussian mixture model [225,226], spectral clustering [227,228], affinity propagation [229], and density-based spatial clustering of applications with noise [230,231] are some of the algorithms that have been used for data anonymization. Most recently, anonymization solutions were proposed for privacy-preserving visual tasks in color images. For instance, the conventional 𝑘-nearest neighbor algorithm was combined with DP [232] for privacy-preserving face attribute recognition and person reidentification. Homomorphic convolution was proposed by combining HE and secret sharing [233] for visual object detection, and adversarial perturbation was devised to prevent disclosure of biometric information in finger-selfie images [234].

##### Models for Synthetic Data

In the study by Choi et al [95], GANs were adopted to generate realistic synthetic patient records (medical GAN [medGAN]; [235]) by learning the distribution of real-world multilabel discrete EHRs. Concretely, medGAN was proposed to generate multilabel discrete patient records through the combination of an autoencoder and a GAN. Such a network supports the generation of both binary and numeric variables (ie, medical codes such as diagnosis, medication, and procedure codes) and the arrangement of records in a matrix format where each row corresponds to a patient and each column represents a specific medical code. The study by Baowaly et al [236] extended the original medGAN by using both Wasserstein GANs with gradient penalty [237] and boundary-seeking GANs [96] to speed up model convergence and stability. In addition, GANs have also been used for segmenting medical images (ie, brain magnetic resonance imaging scans) while coping with privacy protection and data set imbalances [238]. In other words, GANs have proven their potential in data augmentation for imbalanced data sets and data anonymization for privacy preservation. A conditional GAN framework— anonymization through data synthesis-GAN [239]—was proposed to generate synthetic data while minimizing *patient identifiability*, which is based on the probability of reidentification given the combination of all data of any individual patient. In addition, DP has also been used in conjunction with GANs to generate synthetic EHRs [240-243]; most of these models were summarized in a recent survey [244]. On the basis of the CP technique introduced in the previous section, the study by Tseng and Wu [245] presented compressive privacy generative adversarial network to provide a data-driven local privatization scheme for creating compressed representations with lower dimensions for cloud services while removing sensitive information from raw images. Most recently, the conditional identity anonymization GAN [246] was proposed for image and video anonymization based on conditional GANs [247]. Concretely, conditional identity anonymization GAN supports the removal of identifiable information such as characteristics of human faces and bodies while guaranteeing the quality (granularity) of the generated images and videos.

#### Disclosure Risk Assessments

Given the conventional and machine learning–based privacy models, a disclosure risk assessment is usually conducted to measure the reidentification risk of the anonymized EHR data. In practice, risk values from different combinations of privacy models could be used when deciding which version of the anonymized data should be used for data analysis and possible machine learning tasks such as EHR classification with respect to treatment planning or distance recurrence identification.

Concretely, there are 3 major types of disclosure that may occur during the process of data anonymization: identity, attribute, and membership disclosure (Table 3). For practical guidance, we have provided a comparative summary in Multimedia Appendix 6 [248-251] of most of the 35 conventional privacy models investigated (in terms of parameter value ranges and privacy levels).

**Table 3:** Categorization of data reidentification risk metrics for electronic health record data.

Disclosure type and metric		Section in Multimedia Appendix 6	Reference
**Identity**			
	Average risk	1	N/A^a^
	Overall risk	1	N/A
	β-Likeness	1	[125]
	Distance-linked disclosure	2	[248]
**Attribute**			
	Probabilistic linkage disclosure	2	[249]
	Interval disclosure	2	[250]
Membership	Log-linear models	3	[251]

^a^N/A: not applicable.

#### Usability Measurements

The metrics used for measuring the usefulness of the anonymized data can be treated as an on-demand component of a data anonymization system. We revisit the proposed quantitative metrics in this section, although this important indicator is usually not fully covered in the off-the-shelf privacy model–based data anonymization tools. In addition, qualitative metrics are not covered in this study. This is due to the varied objectives of different data anonymization activities, including the evaluation of anonymization quality that is performed by health care professionals. Table 4 lists the selected data usability metrics and the type of data for which they are suitable.

**Table 4:** Categorization of data usability metrics.

Data type and metric			Section in Multimedia Appendix 5		References
**Numerical and categorical**					
	Information loss and its variants	1.1		[172,187-189]	
	Privacy gain	1.2		[190]	
	Discernibility	1.3		[191]	
	Average equivalence class size	1.4		[117]	
	Matrix norm	1.5		[192,193]	
	Correlation	1.6		[194]	
	Divergence	1.7		[195,196]	
**Image^a^**					
	Mean squared error and its variants	2.1		[197-200]	
	Peak signal-to-noise ratio	2.2		[201-206]	
	Structural similarity index	2.3		[207,208]	

^a^Any type of raw and anonymized electronic health record data that can be converted into an image.

### Existing Privacy Model–Based Data Anonymization Tools

**Figure 8:**
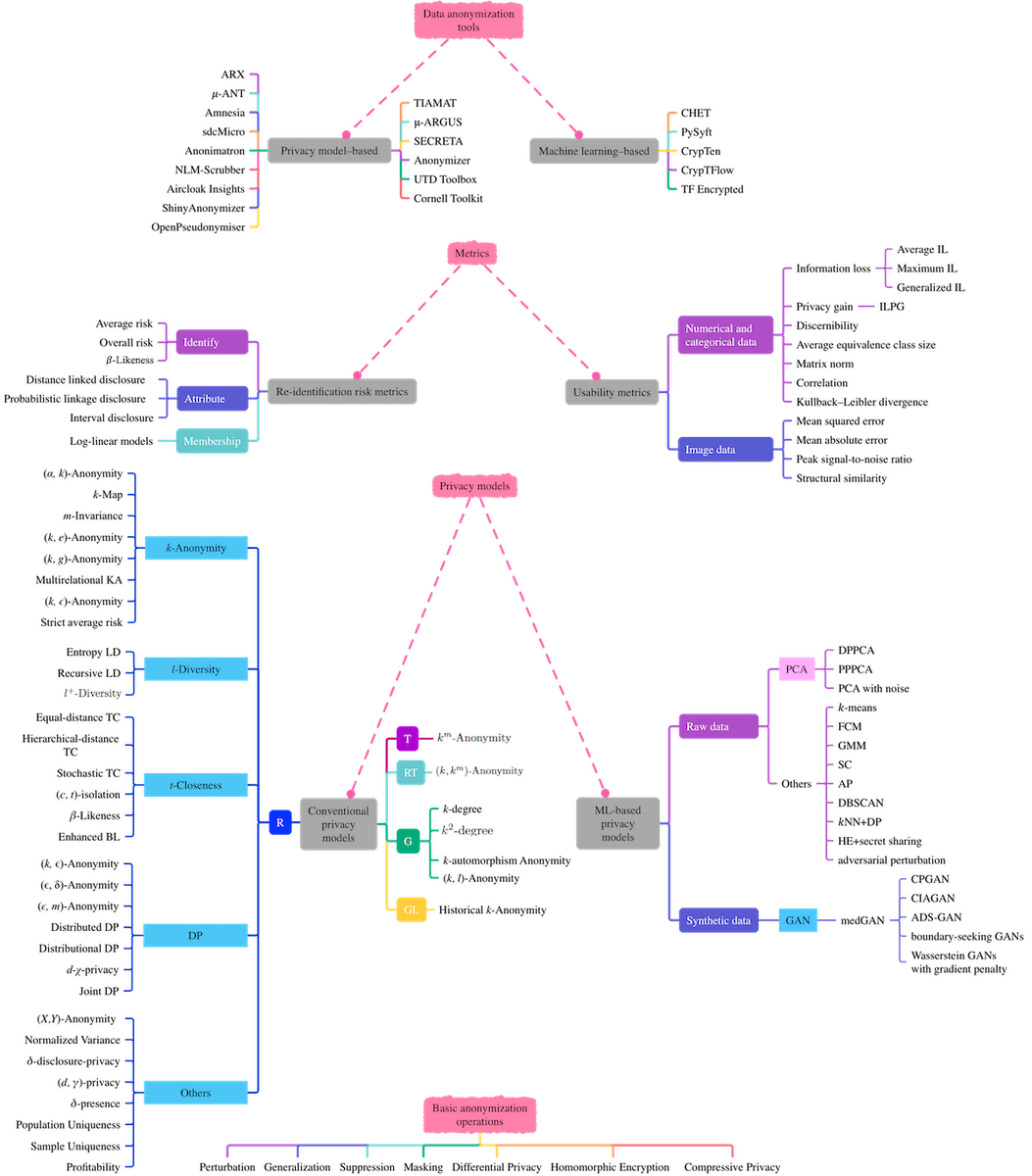
Overall results of the systematic literature mapping study. This mapping consists of four contextually consecutive parts (from bottom to top): basic anonymization operations, existing privacy models, metrics proposed to measure re-identification risk and degree of usability of the anonymized data, and off-the-shelf data anonymization software tools. ADS-GAN: anonymization through data synthesis using generative adversarial networks; AP: affinity propagation; BL: β-Likeness; CIAGAN: conditional identity anonymization generative adversarial network; CPGAN: compressive privacy generative adversarial network; DBSCAN: density-based spatial clustering of apps with noise; DP: differential privacy; DPPCA: differentially private principal component analysis; FCM: fuzzy c-means; G: graph; GAN: generative adversarial network; GL: geolocational; GMM: Gaussian mixture model; HE: homomorphic encryption; IL: information loss; ILPG: ratio of information loss to privacy gain; KA: *k*-Anonymity; *k*NN+DP: *k*-nearest neighbor+differential privacy; LD: *l*-Diversity; medGAN: medical generative adversarial network; ML: machine learning; PCA: principal component analysis; PG: privacy gain; PPPCA: privacy-preserving principal component analysis; R: relational; RT: relational-transactional; SC: spectral clustering; T: transactional; TC: *t*-Closeness.

In this section, several off-the-shelf data anonymization tools based on conventional privacy models and operations are detailed. These tools are commonly adopted for anonymizing tabular data. It should be noted that EHRs are usually organized in the tabular data format and that the real difficulties of anonymizing tabular data lie in the inherent bias and presumption of the availability of limited forecast-linkable data. Therefore, we investigated 14 data anonymization toolboxes, all of which share a similar workflow (summarized in Figure 8 and compared in Table 5 and Table 6). Functionally similar toolboxes are introduced together below.

**Table 5:** Comparison of the off-the-shelf privacy model–based data anonymization tools in terms of available development options, anonymization functionality and risk metrics.

Tool	Last release	Development support						Anonymization		Risk assessment
		Open source	Public API^a^	Extensibility	Cross-platform	Programming language				
ARX	November 2019	✓^b^	✓	✓	✓	Java	✓		✓	
Amnesia	October 2019	✓	✓	✓	✓	Java	✓			
μ-ANT^c^	August 2019	✓	✓	✓	✓	Java	✓			
Anonimatron	August 2019	✓	✓	✓	✓	Java				
SECRETA^d^	June 2019				✓	C++	✓			
sdcMicro	May 2019	✓	✓	Poorly supported	✓	R	✓		✓	
Aircloak Insights	April 2019				✓	Ruby				
NLM^e^ Scrubber	April 2019				✓	Perl				
Anonymizer	March 2019	✓	✓	✓	✓	Ruby				
Shiny Anonymizer	February 2019	✓	✓	✓	✓	R	✓			
μ-ARGUS	March 2018					C++	✓		✓	
UTD^f^ Toolbox	April 2010	✓		Poorly supported	✓	Java	✓			
OpenPseudonymiser	November 2011	✓			✓	Java				
TIAMAT^g^	2009				✓	Java	✓			
Cornell Toolkit	2009	✓		Poorly supported	✓	C++	✓		Poorly supported	

^a^API: application programming interface.

^b^Feature present.

^c^μ-ANT: microaggregation-based anonymization tool.

^d^SECRETA: System for Evaluating and Comparing RElational and Transaction Anonymization.

^e^NLM: National Library of Medicine.

^f^UTD: University of Texas at Dallas.

^g^TIAMAT: Tool for Interactive Analysis of Microdata Anonymization Techniques.

**Table 6:** Comparison of the off-the-shelf privacy model–based data anonymization tools with respect to the supported privacy models.

Tool	Last release	Privacy models									
		*k*-anonymity	*l*-diversity	*t*-closeness	δ-presence	*k*-map	(*k*, *g*)-anonymity	(*k*, ε)-anonymity	(ε, δ)-anonymity	*k^m^*-anonymity	(*k*, *k^m^*)-anonymity
ARX	November 2019	✓^a^	✓	✓	✓	✓			✓		
Amnesia	October 2019	✓									
μ-ANT^b^	August 2019	✓		✓							
Anonimatron	August 2019										
SECRETA^c^	June 2019	✓								✓	✓
sdcMicro	May 2019	✓	✓								
Aircloak Insights	April 2019										
NLM^d^ Scrubber	April 2019										
Anonymizer	March 2019										
Shiny Anonymizer	February 2019										
μ-ARGUS	March 2018	✓									
UTD^e^ Toolbox	April 2010	✓	✓	✓							
OpenPseudonymiser	November 2011										
TIAMAT^f^	2009	✓	✓	✓							
Cornell Toolkit	2009		✓	✓							

^a^Feature present.

^b^μ-ANT: microaggregation-based anonymization tool.

^c^SECRETA: System for Evaluating and Comparing RElational and Transaction Anonymization.

^d^NLM: National Library of Medicine.

^e^UTD: University of Texas at Dallas.

^f^TIAMAT: Tool for Interactive Analysis of Microdata Anonymization Techniques.

Amnesia [252] supports 2 privacy models, *k^m^*-anonymity and *k*-anonymity; the former is used for set-valued and relational-set data sets, and the latter is used for tabular data. Amnesia does not support any reidentification risk assessment; the authors claim that there is no risk associated with the anonymized data set because every query on the anonymized attributes will return at least *k* records.

Anonimatron [253] state that it has been GDPR-compliant since 2010. It supports working with several databases out of the box. It can also be used with text files. The software conducts search-and-replace tasks based on custom rules and as such is merely a pseudonymization tool; however, it is extensible because of its open-source nature.

ARX [164,181,254] was originally developed for biomedical data anonymization. In terms of conventional privacy models, ARX mainly supports 6 additional privacy models: (1) strict average risk, (2) population uniqueness, (3) sample uniqueness, (4) δ-disclosure privacy, (5) β-likeness, and (6) profitability. The population uniqueness can be measured using 4 different models described in the studies by Pitman [148], Zayataz [149], Chen and McNulty [150], and Dankar et al [255]. For *t*-closeness, there are 3 different variants for categorical and numeric data. The profitability privacy model is a game-theoretic model used to conduct cost-benefit analysis and maximize the monetary gains of the data publisher [152]. ARX is open source [256] and supports data of high dimensionality. It is available as a library to be integrated into custom projects or as an installable graphical user interface tool. Similar to ARX, the microaggregation-based anonymization tool (μ-ANT) [257] is also open source [258] and extensible. μ-ANT supports 2 privacy models, *k^m^*-anonymity and *k*-anonymity, as well as *t*-closeness [122]. With respect to usability measurements, μ-ANT supports both information loss and sum of the squared errors. However, μ-ANT does not support functions for filling the missing attribute values (this requires manual data preprocessing, instead, either by removal or filling with average values) or metrics to evaluate the reidentification risk of the anonymized data.

sdcMicro [259] supports 2 privacy models (*k*-anonymity and *l*-diversity) in conjunction with recoding, suppression, postrandomization method (PRAM; which works on categorical data and is usually treated as encompassing noise addition, data suppression, and data recoding. Specifically, each value of a categorical attribute is mapped to a different value in accordance with a prescribed Markov matrix, that is, PRAM matrix), noise addition, and microaggregation. Apart from these functions, this tool also supports the measurement of reidentification risk. As a tool similar to sdcMicro, μ-ARGUS [260] has been implemented in multiple programming languages. It supports anonymization of both microdata and tabular data. It is packaged as disclosure control software and includes *k*-anonymity, recoding (generalization), suppression, PRAM, noise, and microaggregation. Compared with sdcMicro and μ-ARGUS, both University of Texas at Dallas Toolbox [261] and Tool for Interactive Analysis of Microdata Anonymization Techniques [262] support 3 privacy models but lack a risk-assessment module. In addition, University of Texas at Dallas Toolbox was compared with ARX in the study by Prasser et al [263] because of their similar automated anonymization processes and perspectives (in both, the data set is treated as population data, describing one individual per record). In this comparison, ARX showed better performance with respect to execution times and measured data utility.

SECRETA (System for Evaluating and Comparing RElational and Transaction Anonymization) [264] handles 3 categories of data: relational data, transactional data, and relational-transactional data, which are respectively supported by *k*-anonymity and its variants, *k^m^*-anonymity and (*k*, *k^m^*)-anonymity. For relational data sets, SECRETA supports various schemes for data generalization, including full-domain generalization, subtree generalization, and multidimensional generalization. For transactional data, it supports *k^m^*-anonymity using hierarchy-based generalization and constraint-based generalization. For measuring the risk of reidentification, the standalone Identification of Privacy Vulnerabilities toolkit [265] is used.

Aircloak Insights [266,267] can be deemed a data pseudonymization tool because it does not tackle any task of data anonymization. Concretely, by investigating 2 research studies [266,267], we argue that Aircloak Insights is focused more on data protection than on data anonymization. Aircloak Insights comes with a Diffix backend [267], which is essentially a middleware proxy to add noise to user queries for database access in an encrypted fashion. This is also inconsistent with what the authors announced on their official website: “Our privacy-preserving analytics solution uses patented and proven data anonymization that provides GDPR-compliant and high-fidelity insights” [268]. Nevertheless, a number of summarized attacks [267] may be used for validating the efficiency and efficacy of the data anonymization toolbox associated with the Aircloak pipeline.

National Library of Medicine-Scrubber [269] is an anonymization software tool that is specifically designed for coping with unstructured clinical text data. As such, *k*-anonymity is not applicable. Privacy is achieved by applying the HIPAA Safe Harbor model. National Library of Medicine-Scrubber treats text data anonymization as a process of eliminating a specific set of identifiers from the data, and the level of anonymization depends on the comprehensiveness of the identifier lookup data source. In addition, the reidentification risk measurement is not considered in this tool because the authors think that there is no established measure for reidentification of the patient from an anonymized text document.

OpenPseudonymiser [270] and ShinyAnonymizer [271] are very similar: both conduct data encryption only, although they have been specifically designed for medical data. As they only perform data encryption, they are not adequate for data anonymization. Concretely, they support a number of hashing functions (eg, MD5 and SHA512) and encryption algorithms (eg, data encryption standard and advanced encryption standard). Although they support several fundamental data anonymization operations (eg, removing information, suppression, generalization, and bottom and top coding), they do not implement any of the operations in line with privacy models. In addition, they do not provide tools for calculating the risk of reidentification or the measurement of data utility. Similarly, Anonymizer [272] was introduced as a universal tool to create anonymized databases. This tool replaces all data in the given database with anonymized random data where the unique, alphanumeric values are generated by the MD5 hashing function. To this end, the anonymized data might be less useful in view of the authors’ announcement [273]: “There is no way to keep nonanonymized rows in a table”; thus, this software tool is useful for database randomization rather than anonymization.

The Cornell Toolkit [274] supports *l*-diversity and *t*-closeness with flexible parameter configurations. Although the software supports the ability to display the disclosure risk of reidentification of the original tabular data, the method or methods used for implementing the risk measurement have not been introduced in either the paper [274] or in the documentation on the web [275], leaving this software with a low degree of explainability and, hence, trustworthiness.

### Existing Machine Learning–Based Data Anonymization Tools

#### Two Classes

Recently, in response to the GDPR and DPA regulations, efforts were made by the machine learning and cryptography communities to develop privacy-preserving machine learning methods. We define privacy-preserving methods as any machine learning method or tool that has been designed with data privacy as a fundamental concept (usually in the form of data encryption) and that can typically be divided into 2 classes: those that use SMPC and those that use fully HE (FHE). All the investigated machine learning–based data anonymization tools are compared in Table 7.

**Table 7:** Comparison of existing machine learning–based data anonymization tools. The Largest model tested column reports the number of parameters in the largest model shown in the respective tool’s original paper (when reported); CrypTFlow has been shown to work efficiently on much larger machine learning models than the other available privacy-preserving machine learning tools.

Tool	Encryption methods					Reidentification risk assessment		Usability measurement		Development support		
	SMPC^a^	FHE^b^	Differential privacy	Federated learning					Supports training		Malicious security	Largest model tested
CrypTen	✓^c^								✓			N/A^d^
TF Encrypted	✓	✓		✓					✓			419,720
PySyft	✓		✓	✓					✓			N/A
CrypTFlow	✓										✓	65×10^6^
CHET		✓										421,098

^a^SMPC: secure multiparty computation.

^b^FHE: fully homomorphic encryption.

^c^Feature present.

^d^N/A: not applicable.

#### SMPC Frameworks

SMPC involves a problem in which *n* parties, each with their own private input *x_1_, x_2_,..., x_n_* where party *i* has access to input *x_i_* (and only *x_i_*), wish to compute some function *f*(*x_1_, x_2_,..., x_n_*) without revealing any information about their private data [276] to the other parties. Most SMPC frameworks assume the parties to be semihonest: under this scheme we assume that malicious parties still follow the set protocol (although they may work together to attempt to extract private information). The current state-of-the-art framework for SMPC is SPDZ [277], and it is upon this framework that many SMPC-based machine learning libraries are built. This allows data owners to keep their data private and also allows for the machine learning model to be hidden. However, it does require at least three trusted, noncolluding parties or servers to work together to provide the highest level of protection; this can mean it is difficult to implement in practice. There are also significant overheads with this method; not only do SPDZ algorithms necessarily take longer to compute (because of cryptographic overhead), but there is also a significant amount of communication that needs to take place among all participating parties. This results in SMPC machine learning models running approximately 46 times slower than plaintext variants [278], meaning that it is impractical to use such models with large and complex data sets.

There are several different practical implementations of this type of protocol, although none are ready for use in production environments. CrypTen [279] is a library that supports privacy-preserving machine learning with PyTorch. CrypTen currently supports SMPC (although support for other methods such as FHE is in development) by providing SMPC-encrypted versions of tensors and many PyTorch functions; it also includes a tool for encrypting a pre-existing PyTorch model. Although CrypTen supports many of PyTorch’s existing functions, it still has certain limitations. Most notably, it does not currently support graphics processing unit computation, which significantly hinders its ability to be used in conjunction with large, complex models. TensorFlow (TF) Encrypted [280] is a similar framework for the TF open-source software library for machine learning that also supports SMPC through the SPDZ framework. TF Encrypted also includes support for federated learning (which allows the training of machine learning models to be distributed over many devices without each device needing to reveal its private data) and HE.

PySyft [278] is a more general framework than CrypTen or TF Encrypted because it supports multiple machine learning libraries (including TF and PyTorch) and multiple privacy methods. As part of this, it features SMPC-based machine learning, much like CrypTen and TF Encrypted, but also allows for additional layers of security to be incorporated into the model such as DP and federated learning. It is also possible to use TF Encrypted as the provider for TF-based encryption using PySyft, allowing for tighter integration between the 2 libraries. Similar to CrypTen and TF Encrypted, PySyft is a high-level library that attempts to make it easy for machine learning researchers to transition to build privacy-preserving models. However, PySyft should currently only be used as a research tool because many of its underlying protocols are not secure enough to be used with confidence.

CrypTFlow [281] differs from the aforementioned libraries in that it is a compiler for TF models rather than a programming interface. CrypTFlow takes a TF model as an input and outputs code that can run under an SMPC model. An advantage that CrypTFlow has over CrypTen, TF Encrypted, and PySyft is that, as part of its compilation process, CrypTFlow performs a number of optimization steps that in the other libraries would have to be done by hand or cannot be performed at all. For example, when converting floating-point numbers to a fixed-precision representation (which is necessary because SMPC works inside a finite field), CrypTFlow chooses the smallest precision level that will match the classification accuracy of floating-point code. This, along with the other optimizations performed during the compilation process, means that it is possible to (efficiently) run much larger models in CrypTFlow than may be possible in other libraries. The possible real-world impact of CrypTFlow has been shown by running 2 networks designed for predicting lung disease from chest x-rays [282] and diabetic retinopathy [283] from retinal images. It is also possible to use CrypTFlow in conjunction with secure enclaves such as Software Guard Extension 41 (Intel Corporation) to work within the stricter malicious security assumptions; this is stricter than assuming semihonest parties because malicious parties may deviate from the defined protocol. The provision of malicious security means that CrypTFlow is more suitable for use in environments where extreme caution must be taken with the data set being used. Similar to CrypTen, the main issue with CrypTFlow is that it currently does not support the training of machine learning models because it is difficult to use the graphics processing unit in such a setting, meaning that there is still the need to be able to process plaintext data during the training process, which is not compatible with many of the scenarios where one may want to use privacy-preserving machine learning techniques.

An example of how SMPC protocols and SMPC-supporting machine learning libraries can be used is shown in the study by Hong et al [284], which used TF Encrypted to train a classifier on 2 genomic data sets, each containing a large number of features (12,634 and 17,814 features per sample), to detect tumors as part of the iDASH challenge. This task had an additional challenge because the 2 data sets were heavily imbalanced, but common countermeasures to this are difficult to implement in an SMPC framework. For example, resampling is commonly used to overcome this, but because the labels are private in SMPC, this is impossible. To overcome the imbalance, the weighting of samples in the loss function was adjusted to place a higher emphasis on those from the minority class. The study’s best results had an accuracy of 69.48%, which is close to the classifier trained on the plaintext data, which showed an accuracy of 70%. This demonstrates that it is possible to train machine learning models on encrypted data; the study also noted that the TF Encrypted framework is easy to use for anyone familiar with TF, meaning that privacy-preserving machine learning is accessible to experts from both machine learning and cryptography fields.

CrypTen, TF Encrypted, and PySyft all have the advantage that they work closely with commonly used machine learning libraries (PyTorch, TF, and both PyTorch and TF, respectively), meaning that there is less of a learning curve required to make the existing models privacy preserving compared with tools such as CrypTFlow. This ease of use comes at the cost of efficiency, however, because more complex tools such as CrypTFlow are able to work at a lower level and perform more optimizations, allowing larger models to be encrypted.

#### Fully HE

HE is a type of encryption wherein the result of computations on the encrypted data, when decrypted, mirror the result of the same computations carried out on the plaintext data. Specifically, FHE is an encryption protocol that supports any computation on the ciphertext. Attempts have been made to apply FHE to machine learning [285,286]. Traditionally, because of the significant computational overhead required to run FHE computations, these models were trained in plaintext data; for example, it took 570 seconds to evaluate CryptoNet on the Modified National Institute of Standards and Technology data set [285]. It is only recently that we have been able to train a full classification model using FHE computations [36]. The main benefit of FHE over SMPC is that it does not require multiple and separate trusted parties; the models can be trained and run on encrypted data by a single entity. This makes FHE a more promising prospect than SMPC for problems involving data that are too sensitive to be entrusted to multiple parties (or in situations where multiple trusted parties may not be available).

Applying FHE to privacy-preserving machine learning is a relatively new area of research, and thus there are few tools that tie the 2 concepts together, with most research focusing on specific model implementations rather than on creating a general framework for FHE machine learning. One such tool, however, is CHET [287]. CHET is an optimizing compiler that takes a tensor circuit as an input and outputs an executable that can then be run with HE libraries such as Simple Encrypted Arithmetic Library (Microsoft Research) [288] or Homomorphic Encryption for Arithmetic of Approximate Numbers [289]. This automates many of the laborious processes (eg, encryption parameter setting) that are required when creating circuits that work with FHE libraries; these processes also require FHE domain knowledge, which we cannot expect many machine learning experts to possess. Hence, the use of CHET can result in more efficient FHE models. For example, the authors of CHET claim that it reduces the running time for analyzing a particular medical image model (provided by their industry partners) from 18 hours (the original, unoptimized FHE model) to just 5 minutes. However, despite CHET using numerous optimizing methods during its compilation phase, the resulting encrypted models are still restrictively slow (when compared with their nonencrypted counterparts). Not only does this mean it is only practical to use CHET with smaller models, but it also means that it is impractical to train a model using CHET. It is also important to consider whether FHE provides a level of security and privacy that is high enough for the task at hand; some current regulations argue that encryption is a form of pseudonymization rather than anonymization [290] because it is possible to decrypt encrypted data.

### Legal Framework Support

Although general data protection laws such as GDPR and DPA and health care–specific guidelines have been proposed for a while, data anonymization practitioners still demand a combined and intuitive reference list to check. In this discussion, we tentatively construct a policy base by collecting and sorting the available guidance provided by 4 lawful aspects in an effort to benefit future intelligent data anonymization for health care.

The policy base was constructed by considering the documentation provided in accordance with legal frameworks and guidelines proposed by government-accountable institutions, that is, the GDPR, particularly Article 5 [291]; the DPA [292]; the ICO (mainly based on the code of practice); and the NHS (with documents published in 2013 [293], 2015 [294], 2017 [75], 2019 [74,295], and 2021 [296,297]). Fundamentally, any organization (eg, the NHS or a UK company) that holds personal identifiable information is required to register with the ICO, and subsequently perform possible data anonymization followed by a reidentification risk assessment to evaluate the effectiveness of the anonymized data in line with the DPA (the UK implementation of the GDPR). In the case where the NHS or a UK company realizes that a data breach has occurred, it is required to report this to the ICO. In addition, the ICO provides guidance to help the NHS or UK companies to better understand the lawful basis for processing sensitive information. Recently, the ICO [298] and the European Data Protection Board [299] published their statements on the processing of personal identifiable data in coping with the COVID-19 outbreak.

From the NHS perspective, pseudonyms should be used on a one-off and consistent basis. In terms of the best practice recommendations, they recommend adopting cryptographic hash functions (eg, MD5, SHA-1, and SHA-2) to create a fixed-length hash. We argue that the encrypted data might be less useful for possible later data analysis and explainability research. We summarize the suggestions provided by the 4 aforementioned entities in Textbox 2.

**Guidance provided by 4 lawful aspects.**

**General Data Protection Regulation**

- Accuracy
- Accountability
- Storage limitation
- Purpose limitation
- Data minimization
- Purpose limitation
- Lawfulness, fairness, and transparency

**Data Protection Act**

- Notify any personal data breach
- Settle system interruption or restoration
- Implement disclosure-risk measures
- Define legal basis for data processing
- Establish precise details of any processing
- Prevent unauthorized processing and inference
- Conduct data protection impact assessment
- Test anonymization effectiveness through reidentification
- No intent, threaten, or damage to cause in reidentification
- Ensure data integrity when malfunctions occur

**Information Commissioner’s Office**

- Remove high-risk records
- Remove high-risk attribute
- Use average value of each group
- Use the week to replace the exact date
- Swap values of attributes with high risk
- Use partial postcode instead of full address
- Define a threshold and suppress the minority
- Probabilistically perturb categorical attributes
- Aggregate multiple variables into new classes
- Use city instead of postcode and house number, street
- Recode specific values into less-specific range
- Use secret key to link back (data owner only)
- Add noise to numerical data with low variations

**National Health Service**

- Round off the totals
- Swap data attributes
- Use identifier ranges
- Mask part of the data
- Use age rather than date of birth
- Change the sort sequence
- Use the first part of the postcode
- Remove direct identifiers (National Health Service number)
- Risk assessment of indirect identifiers
- Provide only a sample of the population
- Provide range banding rather than exact data
- If aggregate totals less than 5, use pseudonyms

### Results Summary for RQs

Here we present the results of the 5 defined RQs (Textbox 3) and, in the next section, discuss 3 open questions in real-world EHR data anonymization. The overall results of this SLM study are summarized in Figure 7.

**Review questions.**

- Review question (RQ) 1: Do best practices exist for the anonymization of realistic electronic health record (EHR) data?
  As the leading question of this systematic literature mapping study, we answer this question by exploring the answers to the other 4 RQs. It is theoretically feasible but practically challenging. On the basis of the answers to the remaining 4 questions, theoretical operations, privacy models, reidentification risk, and usability measurements are sufficient. Despite this, anonymization is practically difficult mainly because of 2 reasons: (1) the knowledge gap between health care professionals and privacy law (usually requiring huge collaborative efforts by clinical science, law, and data science), although we have summarized all lawful bases in the following subsection; and (2) automatic anonymization of EHR data is nontrivial and very case dependent.
- RQ 2: What are the most frequently applied data anonymization operations, and how can these operations be applied?
  We investigated 7 categories of basic data anonymization operations in 16 articles, most of which are summarized in Multimedia Appendix 3. Apart from their fundamental uses, they can also be incorporated into the data anonymization process in both conventional and machine learning–based privacy models.
- RQ 3: What are the existing conventional and machine learning–based privacy models for measuring the level of anonymity? Are they practically useful in handling real-world health care data? Are there any new trends?
  We presented 40 conventional (a taxonomy for relational data is summarized as part of Figure 7) privacy models and 32 machine learning–based privacy models from a total of 104 articles (summarized as part of Table 1). From this, we have observed that combinations of a deep learning architecture and one or more data anonymization operations have become a trend, particularly techniques based on (conditional-) generative adversarial networks. We have also realized that despite the increasing number of publications from the computer vision community, they rarely use real-world sensitive medical data. For the applicability of existing privacy models, we present an ablation study (Multimedia Appendix 7 [181,300-303]) using publicly accessible EHRs in the next subsection as part of the discussion.
- RQ 4: What metrics could be adopted to measure the reidentification risk and usability of the anonymized data?
  We investigated 7 (from 4 articles) and 15 (from 19 articles) metrics to quantify the risk of reidentification and degree of usability of the anonymized data. Measuring reidentification risk requires a pair of raw and anonymized data records in which the original data are treated as an object of reference and compared with the anonymized data in terms of statistical difference. Such a difference may not sufficiently reveal the true risk of reidentification. To further investigate this issue, we combined the privacy models for discussing the trade-offs between these 2 privacy aspects. In contrast, more usability metrics were proposed because of the wider availability of performance indicators.
- RQ 5: What are the off-the-shelf data anonymization tools based on conventional privacy models and machine learning?
  We investigated and compared 19 data anonymization tools (reported in 36 articles), of which 15 are based on privacy models (compared in Tables 5 and 6), whereas the remaining 5 (compared in Table 7) rely on privacy-preserving machine learning (with issues summarized in the next subsection). However, there does not exist any off-the-shelf data anonymization tool that truly supports the current legal frameworks such as the General Data Protection Regulation and Data Protection Act to dispel the doubts and concerns of data owners (we filled this gap as well).

## Discussion

### Privacy-Usability Trade-offs and Practical Feasibility

The most important question to consider when data anonymization is required in the health care sector is the choice between the level of privacy and degree of usability. In Table 2, we listed parameter interval, which enables specific privacy model or models to be more practically configurable. The privacy level indicates the possible degree of privacy that can be achieved by each privacy model, where separate levels are provided for some variant models such as (α, *k*)-anonymity, stochastic *t*-closeness, and (ε, *m*)-anonymity. This problem can also be viewed as a trade-off between the risk of reidentification and data usability and can be quantified using specific methods [304-306].

It should be noted that the privacy models, reidentification risk measurements, and data usability metrics reviewed in this study are relatively easy to understand, with equations provided along with adequate descriptions. However, these concepts are difficult to deploy in real-world data anonymization tools. Even given the intensive investigations summarized above, the utility of the anonymized data may not be easily measurable through a number of proposed metrics of reidentification risk and utility metrics.

Given this discrepancy observed from the ablation study we conducted (Multimedia Appendix 7), it is worth considering the problem domain when quantifying the reidentification risk as well as the utility of the anonymized data, although we summarized the existing measures in the previous section. Overall, the trade-offs between reidentification risk and usability are practically feasible yet problem dependent.

### Issues of Privacy-Preserving Machine Learning

SMPC and FHE share some disadvantages. They both use encryption methods that work over finite fields, and thus they cannot natively work with floating-point numbers. All practical implementations instead use a fixed-precision representation, but this adds computational overhead, and the level of precision used can affect the accuracy of the results.

Another important issue is that of the trade-off between interpretability and privacy [307] which, where privacy-preserving machine learning is concerned, is highly skewed toward privacy; encrypted models are, because of their very nature, entirely black-box models. This is not only an issue in the health care field, where the explainability of machine learning models is an important issue [308], but also arguably in any machine learning application because of the GDPR’s “right to an explanation” [309].

Encrypted, trained models are also still vulnerable to reverse-engineering attacks (regardless of the encryption method used) [278]; for example, a malicious user could use the outputs of a model to run a membership attack (ie, infer from the results of a model whether the input was from a member of the training set). Currently, the only known way to overcome this is to apply DP principles to the model, which adds yet another layer of complexity to the process. There are signs that existing libraries are starting to combat the possibility of such attacks by providing easy methods to apply DP to encrypted models; see, for example, the DP techniques available in PySyft in the *SMPC Frameworks* section above.

It is also important to remember that, as noted previously, any type of encryption is regarded as a form of pseudonymization rather than anonymization because the encrypted data can be decrypted by anyone with access to the encryption key. However, we note that much of the current guidance on viewing encryption techniques as anonymization or pseudonymization is ambiguous; for example, ICO guidance [290] suggests that encrypted data is classified as anonymized data so long as the party responsible for the encryption of the personal data is not also responsible for the processing of the encrypted data (because then the party processing the data would not be in possession of the encryption key and would therefore be unable to reverse the encryption). As such, it is important to carefully consider whether privacy-preserving machine learning techniques fully satisfy the requirements set out in law. For instance, tools that also include other privacy techniques, such as PySyft, may be more useful in situations where true anonymization is required.

Overall, privacy-preserving machine learning is a promising area of research, although more work needs to be undertaken to ensure that such methods are ready for use in industrial applications; many of the tools currently available are only suitable for research rather than practical application. There also needs to be some consideration over which privacy-preserving methods best suit the needs of the application. SMPC currently offers a more viable approach than FHE because of its ability to run (and, more importantly, train) larger models, although the need to have multiple trusted parties may mean that it is seen as less secure than FHE. Meanwhile, FHE for privacy-preserving machine learning is still an emerging field, and it is encouraging to see research being undertaken by both the machine learning and cryptographic communities to improve the practicality of FHE methods by improving the running time of encrypted models and reducing the level of cryptographic knowledge needed to create efficient, encrypted models using FHE.

### Conclusions

In this SLM study, we presented a comprehensive overview of data anonymization research for health care by investigating both conventional and emerging privacy-preserving techniques. Given the results and the discussions regarding the 5 proposed RQs, privacy-preserving data anonymization for health care is a promising domain, although more studies are required to be conducted to ensure more reliable industrial applications.

## Appendix

Multimedia Appendix 1 Math notation system.

Multimedia Appendix 2 Typical direct identifiers and quasi-identifiers in UK electronic health record data.

Multimedia Appendix 3 Examples of fundamental data anonymization operations.

Multimedia Appendix 4 Conventional privacy models.

Multimedia Appendix 5 Usability metrics for privacy models.

Multimedia Appendix 6 Reidentification risk metrics for privacy models.

Multimedia Appendix 7 Ablation study for privacy-usability trade-offs and practical feasibility.

## Abbreviations

CP: compressive privacy
DP: differential privacy
DPA: Data Protection Act
EHR: electronic health record
FHE: fully homomorphic encryption
GAN: generative adversarial network
GDPR: General Data Protection Regulation
GP: general practitioner
HE: homomorphic encryption
HIPAA: Health Information Portability and Accountability Act
ICO: Information Commissioner’s Office
medGAN: medical generative adversarial network
NHS: National Health Service
PCA: principal component analysis
PRAM: postrandomization method
QI: quasi-identifier
RQ: review question
SECRETA: System for Evaluating and Comparing RElational and Transaction Anonymization
SLM: systematic literature mapping
SLR: systematic literature review
SMPC: secure multiparty computation
TF: TensorFlow
μ-ANT: microaggregation-based anonymization tool
