# Supplementary material for: Data Anonymization for Pervasive Health Care: Systematic Literature Mapping Study
Source: JMIR Med Inform. 2021 Oct 15;9(10):e29871. doi: 10.2196/29871 (PMC8556642; doi:10.2196/29871)
Supplement: Multimedia Appendix 1 [file medinform_v9i10e29871_app1.pdf]

Multimedia Appendix 1

Math notation system

Table 1: Symbols and definitions.

$\mathbf{X}$	Raw data	$\mathbf{X}^{'}$	Anonymized data
S	Sensitive attributes	Q	Quasi-identifiers[a]
$\mathcal{E}$	Equivalent class, $\dot{\mathcal{E}} \in \mathcal{E}$	N	Numerical attribute, $N \in Q$
C	Categorical attribute	A	Privacy mechanism (or function)
$\dot{H}$	A pre-defined hierarchy	$\mathcal{P}$	A set of patients $p \in \mathcal{P}$
$\mathcal{L}$	Level of anonymity	$\mathcal{R}$	Risk measurement
$\mathcal{U}$	Usability metric	f	A probability distribution
$\mathcal{I}$	A Boolean indicator	$\underline{z}$	Minimum value
$\bar{z}$	Maximum value	γ	Weights
$\mathcal{H}(\cdot)$	Entropy	$\texttt{dist}(\cdot)$	Distance
$\wedge$	T-norm operator[b]	$\bowtie$	Outer join operation

[a]Quasi-identifiers are also known as indirect identifiers.
[b]A t-norm operator usually implemented as a minimum operator.
