# Supplementary material for: Data Anonymization for Pervasive Health Care: Systematic Literature Mapping Study
Source: JMIR Med Inform. 2021 Oct 15;9(10):e29871. doi: 10.2196/29871 (PMC8556642; doi:10.2196/29871)
Supplement: Multimedia Appendix 2 [file medinform_v9i10e29871_app2.pdf]

Multimedia Appendix 2

Typical direct identifiers and quasi-identifiers in UK EHR data. EHR: electronic health record

Table 2: Direct I and quasi- Q identifiers for mainly UK-based electronic health records (EHR) data.

**Category**	**Identifier**	**Comments/Examples**
I	Names	Full name, nickname, initials (patient and relatives)
	Geographical information	Current/past subdivisions (city/county), full/partial post code
	Date of birth (DoB)	Day/month/year
	(Contact) numbers	Mobile/tel./fax/NI, BRP, (provisional) driving licence
	(Contact) address	Current and previous living/email/IP/URL
	Health-related information	Medical records/plans, NHS number
	Accounts	Apple, Google, Microsoft, Instagram, etc.
	Vehicle-related information	Car licence plate, serial number, insurance record
	Device-related information	Serial number (phone/laptop/tablet/desktop)
	Biometric-related information	Finger, retina, voice prints
	Full face images	And any comparable ones
	Unique booking no.	Booking reference number with a NHS trusted hospital
	Any other unique identifiers	QR code, handwritten/digital signature, date of death
Q	Gender	Especially for rare disease
	General birth information	Year of birth, age
	Places	Place of birth/health professional responsible for care[b]
	Socioeconomic data	Occupation, income, education
	Anthropometry measures[a] [72]	Height, weight, Body Mass Index (BMI), A1c
	Ethnicity and nationality	Ethnic origin and country
	Risky behaviour	Illicit drug use, drug dependence
	Pregnancy information	Especially for multiple pregnancies
	Small denominators [71]	Population size less than 100
	Very small numerators [71]	Event count less than 3

[a]Refer to MRC explanations for more details.
[b]Refer to MRC guidance for more details.
