# Supplementary material for: Data Anonymization for Pervasive Health Care: Systematic Literature Mapping Study
Source: JMIR Med Inform. 2021 Oct 15;9(10):e29871. doi: 10.2196/29871 (PMC8556642; doi:10.2196/29871)
Supplement: Multimedia Appendix 3 [file medinform_v9i10e29871_app3.pdf]

# Multimedia Appendix 3

## Examples of fundamental data anonymization operations

Table 3: A comprehensive revisit of fundamental data anonymisation operations.

<table>
<tr><th>Method</th><th>Raw data</th><th>Processed data</th></tr>
<tr><td>Micro-aggregation [101]</td>
<td><table><tr><th>Dist. Hosp.</th><th>Dist. Bus/Metro</th></tr><tr><td>4.4 km</td><td>0.5 km</td></tr></table></td>
<td><table><tr><th>Aggregate Dist.</th></tr><tr><td>4.9 km</td></tr></table></td></tr>
<tr><td>Data swapping [102]</td>
<td><table><tr><th>Name</th><th>Age</th><th>Place of Birth</th></tr><tr><td>Luke</td><td>36</td><td>Berkshire</td></tr><tr><td>Jason</td><td>44</td><td>Yorkshire</td></tr><tr><td>Elvis</td><td>72</td><td>Derbyshire</td></tr></table></td>
<td><table><tr><th>Name</th><th>Age</th><th>Place of Birth</th></tr><tr><td>Luke</td><td>72</td><td>Yorkshire</td></tr><tr><td>Jason</td><td>36</td><td>Derbyshire</td></tr><tr><td>Elvis</td><td>44</td><td>Berkshire</td></tr></table></td></tr>
<tr><td>Post-randomization [105]</td>
<td><table><tr><th>Name</th><th>Age</th><th>Place of Birth</th></tr><tr><td>Luke</td><td>36</td><td>Berkshire</td></tr><tr><td>Jason</td><td>33</td><td>Berkshire</td></tr><tr><td>Elvis</td><td>37</td><td>Berkshire</td></tr></table></td>
<td><table><tr><th>Name</th><th>Age</th><th>Place of Birth</th></tr><tr><td>Luke</td><td>36</td><td>Berkshire</td></tr><tr><td>Jason</td><td>55</td><td>Yorkshire</td></tr><tr><td>Elvis</td><td>37</td><td>Berkshire</td></tr></table></td></tr>
<tr><td>Adding noise [106]</td>
<td><table><tr><th>Age</th></tr><tr><td>54</td></tr></table></td>
<td><table><tr><th>Age</th></tr><tr><td>61</td></tr></table></td></tr>
<tr><td>Resampling [107]</td>
<td><table><tr><th></th><th>Age</th><th>Dist. Bus</th><th>Dist. Hosp.</th></tr><tr><td>2019-01-01</td><td>45</td><td>2.917872</td><td>5.546464</td></tr><tr><td>2019-01-04</td><td>43</td><td>2.279478</td><td>6.810556</td></tr><tr><td>2019-01-07</td><td>53</td><td>1.960084</td><td>7.146709</td></tr><tr><td>2019-01-10</td><td>29</td><td>1.142433</td><td>1.785064</td></tr><tr><td>2019-01-13</td><td>54</td><td>1.229839</td><td>3.624859</td></tr></table></td>
<td><table><tr><th></th><th>Age</th><th>Dist. Bus</th><th>Dist. Hosp.</th></tr><tr><td>2019-01-31</td><td>46</td><td>1.418458</td><td>4.678576</td></tr><tr><td>2019-02-28</td><td>42</td><td>1.224011</td><td>3.382083</td></tr><tr><td>2019-03-31</td><td>35</td><td>1.267175</td><td>5.703625</td></tr><tr><td>2019-04-30</td><td>35</td><td>1.069460</td><td>4.935450</td></tr><tr><td>2019-05-31</td><td>33</td><td>1.430751</td><td>3.689995</td></tr></table></td></tr>
<tr><td>Generalization [108]</td>
<td><table><tr><th>Postcode</th></tr><tr><td>DH1 3LE</td></tr></table></td>
<td><table><tr><th>Postcode</th></tr><tr><td>DH1</td></tr></table></td></tr>
<tr><td>Data masking [110]</td>
<td><table><tr><th>Debit card no.</th></tr><tr><td>9990 0305 2002 2100</td></tr></table></td>
<td><table><tr><th>Debit card no.</th></tr><tr><td>**** **** **** *100</td></tr></table></td></tr>
<tr><td>Differential privacy [111]</td>
<td><table><tr><th></th><th>Age</th><th>Postcode</th></tr><tr><td>0</td><td>22</td><td>NE3 2TL</td></tr><tr><td>1</td><td>24</td><td>NE4 1XE</td></tr><tr><td>2</td><td>22</td><td>NE6 9PO</td></tr><tr><td>3</td><td>25</td><td>NE1 4YU</td></tr></table></td>
<td><table><tr><th></th><th>Age</th><th>Postcode</th></tr><tr><td>0</td><td>22</td><td>NE3 2TL</td></tr><tr><td>1</td><td>24</td><td>CE4 5JK</td></tr><tr><td>2</td><td>22</td><td>NE6 9PO</td></tr><tr><td>3</td><td>25</td><td>SE4 3LC</td></tr></table></td></tr>
</table>
