# Supplementary material for: Data Anonymization for Pervasive Health Care: Systematic Literature Mapping Study
Source: JMIR Med Inform. 2021 Oct 15;9(10):e29871. doi: 10.2196/29871 (PMC8556642; doi:10.2196/29871)
Supplement: Multimedia Appendix 4 [file medinform_v9i10e29871_app4.pdf]

Multimedia Appendix 4
Conventional privacy models

1 Privacy Models for Relational Data

1.1 k-Anonymity

The k-anonymity (KA) model anonymizes data by generalizing quasi-identifiers, ensuring that an individual's data is indistinguishable from at least $(k-1)$ others' [118, 119]. This can achieved by either a combination of generalization and local suppression [118], or via micro-aggregation [120]. However, the process of finding the optimal k poses an NP-hard problem [121]. We focus on three k-anonymity algorithms: Datafly, Incognito, and Mondrian.

$$\mathcal{L}_{\mathrm{KA}} = k, \quad \text{s.t. } \forall \mathcal{E} : |\mathcal{E}| \geqslant k. \tag{1}$$

Datafly, a greedy heuristic algorithm, can be employed to anonymize a table and satisfy k-anonymity [122]. Incognito is a single-dimensional full-domain generalization algorithm that builds a generalization lattice and traverses it using a bottom-up breadth-first search [123]. Mondrian is a greedy multidimensional algorithm that partitions the domain space recursively into several regions, each of which contains at least k records [124]. A comparative study has been conducted in the research work of [125] in line with the same anonymization operations, generalization and suppression.

However, k-anonymity suffers from the well known *curse of dimensionality*. That is, as the scale of the data increases the space- and time-requirements for the algorithm increase exponentially. In addition, k-anonymity is vulnerable against homogeneity and background knowledge attacks [126]. In the homogeneity attack, the adversary can infer sensitive attributes of a data record if sensitive values within an equivalent class lack diversity. In the background knowledge attack, the adversary can achieve the same goal when background knowledge is available, even if the anonymized data is adhering to k-anonymity.

1.1.1 (α, k)-Anonymity

It has been proven that k-anonymity is NP-hard when using minimum generalization with $k \geqslant 2$ [121]. In addition, k-anonymity does not constrain sensitive attributes when most records share the same sensitive attribute values in the settings of high dimensions and multi-constraints. This naturally results in a high probability of inferring sensitive information from the perspective of attackers. To this end, (α, k)-anonymity [127] is achieved by ensuring the k-anonymity is satisfied while the records related to the value of any sensitive attributes in each equivalent class are less or equal to $1/\alpha$.

$$\mathcal{L}_{\mathrm{AKA}} = (\alpha, k), \quad \text{s.t. } \forall \mathcal{E} : \frac{|(\mathcal{E}, S)|}{|\mathcal{E}|} \leqslant \alpha \wedge |\mathcal{E}| \geqslant k. \tag{2}$$

1.1.2 k-Map

The k-map (KM) privacy model is related to k-anonymity, but risk is calculated based on information about the underlying population via generalization and suppression [119,128]. Generally, it was designed based on the hypothesis that the data controller can k-anonymize the identification database itself, and can thereby

control the risk of re-identification directly. The objective is to determine whether k-anonymity is satisfied on an identification database when combined with the database being anonymized (assuming the attacker has access to both). If it is not satisfied then changes should be applied to rows in the actual database that satisfy the model on the data as a whole. This technique may be useful for particularly small data sets where the application of k-anonymity would compromise data richness. However, KM is not frequently employed in the practical data anonymization process, as it assumes that the data controller does not have access to the identification (population) database, but that an intruder does [119]. k-anonymity is more practically feasible compared to KM as in the former data owners, or sometimes processors, secure what they have access to and can focus on the requirements of the data set at hand.

1.1.3 m-Invariance

m-Invariance (MI) enhances k-anonymity by supporting multiple variations of the same dataset with added, modified, or removed data records (*i.e.* rows). Given multiple releases of the anonymized data, it is achievable that the attackers can infer the values of the sensitive attributes. To cope with this possible attack, m-invariance requires that each equivalence class $\mathcal{E}$ must contain at least m rows, and the values for sensitive attributes S must be completely different [129]. Additionally, the set of unique sensitive values in each $\mathcal{E}$ must be consistent in each release.

$$\begin{aligned} & \mathcal{L}_{\mathrm{MI}} = m \\ \text{s.t.} \quad & \forall \mathcal{E} : |\mathcal{E}| \geqslant m, \\ & \forall S_i, S_j \in \mathcal{E} : S_i \neq S_j, \\ & \forall \mathcal{E} : \text{number of unique } S \text{ must be consistent in all the published versions.} \end{aligned} \tag{3}$$

1.1.4 (k, e)-Anonymity

The (k, e)-Anonymity (KEA) privacy model [130] enhances k-anonymity to apply to numerical attributes instead of categorical ones by additionally requiring that the range of the sensitive attributes S in any equivalent class $\mathcal{E}$ to be greater than e.

$$\mathcal{L}_{\mathrm{KEA}} = (k, e), \quad \text{s.t. } \forall \mathcal{E} : |\mathcal{E}| \geqslant k \wedge |\bar{\mathcal{E}} - \underline{\mathcal{E}}| > e. \tag{4}$$

It has been proven that KEA suffers from proximity attacks [131] with respect to attribute disclosure. This is mainly due to the fact that KEA is not strict on the distribution of the values in the range e. Concretely, suppose that the majority of the sensitive values are distributed in a short interval within e, then the attackers are able to infer membership with high degree of confidence [57].

1.1.5 (k, g)-Anonymity

(k, g)-Anonymity (KGA) privacy model [132], which enhances the original k-anonymity model, was proposed for efficiently capturing the diversity and dispersibility of sensitive numerical values in each equivalence class. This model was achieved using the Maximum Distance to Average Vector (MDAV) algorithm. MDAV [133] is a useful micro-aggregation algorithm for numerical data. In general, by meeting k-anonymity, (k, g)-anonymity requires that the balanced adjacent degree ($\mathtt{dist}$) between the global distribution S and equivalent class distribution $S_{\mathcal{E}}$ is greater than a constant $g \in [0, 1]$.

$$\mathcal{L}_{\mathrm{KGA}} = (k, g), \quad \text{s.t. } \forall \mathcal{E} : |\mathcal{E}| \geqslant k \wedge \mathtt{dist}(S, S_{\mathcal{E}}) > g. \tag{5}$$

1.1.6 Multirelational k-Anonymity

Multirelational k-Anonymity (MKA) [134] enhances k-anonymity by extending the definition of k-anonymity to relational databases. This is achieved by joining the database table identifying record owners $\mathbf{X}_{\mathrm{master}}$ with all the tables that contain database records $\mathcal{X}_i$, and then feed the result of the join $\mathcal{J}$ into k-anonymity. For

each record owner in $\mathbf{X}_{\text{master}}$, $\mathcal{J}$ must contain at least $k-1$ other record owners with the same quasi-identifier values Q, thereby the equivalent classes $\mathcal{E}_{\text{master}}$ contain all record owners with the same Q, rather than all the records with the same Q as in original k-anonymity.

$$\mathcal{L}_{\text{MKA}} = k, \quad \text{s.t. } \mathcal{J} = \mathbf{X}_{\text{master}} \bowtie \mathcal{X}_1 \bowtie \cdots\cdots \mathcal{X}_n \wedge \forall \mathcal{E}_{\text{master}} \in \mathcal{J} : |\mathcal{E}_{\text{master}}| \geqslant k. \tag{6}$$

1.1.7 Strict Average Risk

Fundamentally, average risk[1] is defined to prevent re-identification using a threshold on the risk of each record in the raw data $\mathbf{X}$ [A4_1]. It is commonly assumed that no record in $\mathbf{X}$ is more vulnerable than the rest ones to a re-identification attach due to the limited data access. In this case, average re-identification probability across all the records is adopted to measure the re-identification risk. Furthermore, in order to protect against the unique records or equivalence classes with a high re-identification risk, the strict average is required where no record is with a re-identification risk greater than a specified threshold. Obviously, strict average is proposed to guarantee that there are no unique records and that the average re-identification risk is acceptably low. Thus, strict average risk [135] is a combination of average risk and k-anonymity.

1.2 l-Diversity

Due to the fact that k-anonymity is redundant against background knowledge and homogeneity attacks, l-Diversity (LD) was proposed to ensure each k-anonymous group contains at least l different values of the sensitive attribute [126]. LD is used to prevent attribute disclosure by ensuring sensitive attribute values in each equivalence class (EC) are diverse, *i.e.* requiring at least l values in each equivalent class. However, LD is unable to prevent attribute disclosure against skewness and similarity attacks [137]. That is, similarity attacks are possible when attribute values are different yet structurally similar within an equivalent class, and a skewness attack occurs when l-diversity does not prevent attribute disclosure. To this end, LD can be instantialized to entropy LD (ELD) and recursive LD (RLD). In ELD, the sensitive values in each EC should be different enough and evenly distributed enough, *i.e.* the entropy of the distribution of sensitive values in each equivalent class $\mathcal{H}(S_{\mathcal{E}})$ is at least $\log(l)$. In RLD, the most common values should appear frequent enough denoted as s_i while less common ones should not appear too infrequently that controlled by a constant γ.

$$\begin{cases} \mathcal{L}_{\text{ELD}} = l, & \text{s.t. } \forall \mathcal{E} : \mathcal{H}(S_{\mathcal{E}}) \geqslant \log(l) & \text{Entropy LD,} \\ \mathcal{L}_{\text{RLD}} = l, & \text{s.t. } \forall \mathcal{E} : s_1 < \gamma(s_l + s_{l+1} + \cdots + s_n) & \text{Recursive LD.} \end{cases} \tag{7}$$

1.2.1 l^+-Diversity

It is well-known that l-diversity is proposed to enhance the k-anonymity by preventing homogeneity attacks, that is, there are at least l values for a given sensitive attribution s_i within each indistinguishable group of records. However, l-diversity is NP-hard [121,124]. l^+-Diversity [138] is proposed by assigning a different privacy threshold to each s_i for reducing the information loss. Concretely, this privacy model requires no individual in the anonymized data $\mathbf{X}^{'}$ can be linked to a s_i with a probability higher than a crisp threshold θ_i, which can be denoted as $\Pr[s_i|q_i^{'}] \leq \theta_i$, s.t. $s_i \in S, q_i^{'} \in \mathcal{Q}^{'}$, and $\theta_i = \frac{1}{l} \in [0,1]$. This model can be achieved by generalization and suppression.

1.3 t-Closeness

t-Closeness (TC) is an enhancement to l-diversity to preserve privacy in datasets by reducing the granularity of a data representation [139]. It can be viewed as the distance among the distribution of a sensitive attribute within any class, and the distribution of the attribute in the entire table is less than or equal to a threshold t. The distance `dist`$(\cdot)$ is calculated using earth mover distance (EMD).

[1]`https://www.lexjansen.com/phuse/2016/dh/DH09.pdf`

$$\mathcal{L}_{\mathrm{TC}} = t, \quad \text{s.t. } \forall \mathcal{E} : \texttt{dist}(S, S_{\mathcal{E}}) \leqslant t. \tag{8}$$

In practice, TC can be integrated with Equal-Distance EMD (EDTC), Hierarchical-Distance EMD (HDTC), and Ordered-Distance EMD (ODTC) [139]. The former requires the specification of the sensitive attribute and value of t, whereas the latter requires additional at least one hierarchy for sensitive attribute *e.g.* 'Postcode'. In addition, TC could also be combined with k-anonymity in microaggregation to achieve a trade-off between protection of attribute disclosure and data utility [140].

1.3.1 Stochastic t-Closeness

The values of sensitive attributes were kept intact in the original version of t-Closeness. In contrast, stochastic t-closeness (STC) [141] is proposed for modifying sensitive values randomly. Additionally, STC can be employed to bridge the gap between k-anonymity and differential privacy, where the former is used for anonymising the data and latter is adopted for limiting the knowledge gain of the attacker. It is also noteworthy that, if the distribution of S satisfies ϵ-differential privacy, the data table will satisfy STC, in which the values of t are not only dependent on the given data tables but also ϵ.

1.3.2 (c, t)-Isolation

(c, t)-Isolation (CTI), as an extension of k-anonymity, is proposed to measure how well the attacker(s) are able to isolate points in the real database $\mathbf{X}$ [142]. The difference between attacker's estimation $\hat{x}$ and group truth x is denoted as $\Delta_{\hat{x}}$. If the attacker's estimation matches the ground truth, then x is (c, t)-isolated. That is, a ball $\mathcal{B}$ with radius r equals to $c\Delta_{\hat{x}}$ around the attacker's estimation includes fewer than t other points, and c is an isolation parameter for controlling the size of the ball and t is the privacy threshold.

$$\mathcal{L}_{\mathrm{CTI}} = (c, t), \quad \text{s.t. } |\mathcal{B}(\hat{x}, c\Delta_{\hat{x}}) \cap \mathbf{X}| < t \wedge \Delta_{\hat{x}} = \|\hat{x} - x\|. \tag{9}$$

1.3.3 β-Likeness and Enhanced β-Likeness

β-Likeness (BL) is closely related to t-closeness (Section 1.3) and δ-disclosure privacy (Section 1.5.3). It can also be used to protect from possible attribute disclosure for data anonymization, particularly for microdata [143].

$$\mathcal{L}_{\mathrm{BL}} = \max\left(\texttt{dist}\Big(S, S_{\mathcal{E}}\Big)\Big| s \in S, e \in \mathcal{E}, s < e\right) \leqslant \beta. \tag{10}$$

However, BL suffers from the possible record level re-identification with respect to the value of sensitive attributes S within each equivalent class $\mathcal{E}$ with high degree of confidence. In addition, the degree of confidence heavily relies on the frequency of S values. For resolving this issue, the Enhanced β-Likeness (EBL) is proposed by adding the additional constraint to S via:

$$\mathcal{L}_{\mathrm{EBL}} = \max\left(\texttt{dist}\Big(S, S_{\mathcal{E}}\Big)\Big| s \in \mathcal{S}, e \in \mathcal{E}, s < e\right) \leqslant \min(\beta, \ln S). \tag{11}$$

1.4 Differential Privacy

Differential Privacy (DP) [111] is a constraint on the algorithms (*e.g.* privacy models) used to share or publish aggregated information about a dataset which maximises the privacy preservation of the information in the dataset. DP is proposed to ensure that any privacy disclosure is unlikely to happen without considering whether a specific term exists in the database or not.

1.4.1 (k, ϵ)-Anonymity

ϵ-Differential Privacy [144] (EDP), as the most typical DP algorithm, was defined for the privacy loss associated with any data record or the entire dataset. It can be defined as:

$$\mathcal{L}_{\text{EDP}} = \forall S \subseteq \text{Range}(A) : \Pr[A(\mathcal{X}_1) \in S \in \mathcal{X}] \leqslant \exp(\epsilon) \times \Pr[A(\mathcal{X}_2) \in S \in \mathbf{X}], \tag{12}$$

where A is a randomised algorithm (or a function) supporting ϵ-differential privacy, S is the sensitive attribute in the data $\mathbf{X}$, $\mathcal{X}_1$ and $\mathcal{X}_2$ are neighbouring subsets of database $\mathbf{X}$ s.t. $|\mathcal{X}_1 - \mathcal{X}_2|_1 \leq 1$ (*i.e.* the Hamming distance between them is at most one patient's data), *i.e.* $\exp(\epsilon), \epsilon \in_0^+$. However, it is difficult to determine the value of ϵ in real-world domains [145,146,147]. Based on ϵ-differential privacy, (k, ϵ)-anonymity was proposed by [148], which was implemented by the IBM Differential Privacy Library[2] [149].

1.4.2 (ϵ, δ)-Anonymity

(ϵ, δ)-Anonymity (EDA), which is also known as approximate differential privacy, was proposed in [150,151] by adding an additional additive coefficient δ. EDA is designed for pursuing a higher degree of usability and reducing the level of anonymity than DP, by tolerating larger categories of queries [152], reducing the sample complexity of private learning [153]. In addition, EDA requires δ to be smaller than the inverse of any polynomial taken in the size of $\mathbf{X}$ [154], *i.e.* $\frac{1}{\|\mathbf{X}\|}$ where δ is usually valued within the interval of $[10^{-5}, 1]$ [155]. This naturally results in a smaller number of records to be published within the realm of DP. In practice, a data generalization scheme is usually created for flexibly specifying the degree of generalization.

$$\mathcal{L}_{\text{EDA}} = \forall S \subseteq \text{Range}(A) : \Pr[A(\mathcal{X}_1) \in S \in \mathcal{X}] \leqslant \exp(\epsilon) \times \Pr[A(\mathcal{X}_2) \in S \in \mathbf{X}] + \delta. \tag{13}$$

1.4.3 (ϵ, m)-Anonymity

The (ϵ, m)-Anonymity (EMA) privacy model [131], as another extension of k-anonymity, is proposed for addressing proximity attacks by bounding the probability of inferring sensitive values to at most $1/m$. This is achieved by limiting the number of elements in the equivalent class $\mathcal{E}$ with numerically ϵ-similar sensitive values S.

$$\begin{aligned} &\mathcal{L}_{\text{EMA}} = \forall \mathcal{E} : \forall e \in \mathcal{E} : \left|\frac{\tilde{\mathcal{E}}}{\mathcal{E}}\right| \leqslant \frac{1}{m} \\ \text{s.t.}\quad &\tilde{\mathcal{E}} \text{ denotes the members of } \mathcal{E} \text{ whose } S \text{ are with the range of } [S(e) - \epsilon, S(e) + \epsilon]. \end{aligned} \tag{14}$$

1.4.4 Distributed Differential Privacy

Distributed Differential Privacy (DDP) [156] extends (ϵ, δ)-anonymity by setting how data from distributed entities reaches a central data aggregator. This model allows a level of data randomization to be performed when participants (or patients) do not trust the data aggregator to which the data is sent. Essentially, this privacy model allows a higher degree of flexible privacy control, *i.e.* a group of patients $\dot{\mathcal{P}}$ is a subset of $\mathcal{P}$ that are able to collude with the data aggregator while retaining certain privacy guarantees for the remaining patients. In this case, the probability in differential privacy is conditioned by a random factor that is given by comprised patients.

$$\mathcal{L}_{\text{DDP}} = \forall S \subseteq \text{Range}(A), \dot{\mathcal{P}} \subset \mathcal{P} : \Pr[\mathcal{A}(\mathcal{X}_1) \in S|_{r_{\dot{\mathcal{P}}}} \in \mathcal{X}] \leqslant \exp(\epsilon) \times \Pr[A(\mathcal{X}_2) \in S|_{r_{\dot{\mathcal{P}}}} \in \mathbf{X}] + \delta. \tag{15}$$

[2] `https://github.com/IBM/differential-privacy-library`

1.4.5 Distributional Differential Privacy

Distributional Differential Privacy (DisDP) [157] extends differential privacy to cope with the situation where the datasets do not require protection, but instead the parameters (θ_1 and θ_2) to control the generation of data. Concretely, given parameters θ_1 and θ_2 for generating two datasets containing at most one different element. The anonymization mechanism A satisfies the distributional ϵ-differential privacy in the case where the query response A_i is similarly generated using θ_1 and θ_2.

$$\mathcal{L}_{\text{DisDP}} = \Pr[\theta_1|A_i] \leqslant \exp(\epsilon) \times \Pr[\theta_2|A_i]. \tag{16}$$

1.4.6 d-χ-Privacy

d-χ-Privacy (DChiP) [158] is a generalization of differential privacy which utilizes an alternative distinguishability metric d_χ to measure the distance between datasets $\mathcal{X}_1$ and $\mathcal{X}_2$, instead of using Hamming distance (*i.e.* the distance 1 is ϵ) employed in original DP. Essentially, DChiP can achieve different privacy concepts by working jointly with different distance metrics. For instance, Euclidean distance can be considered for geolocation information in the healthcare datasets.

$$\mathcal{L}_{\text{DChiP}} = \texttt{dist}[A(\mathcal{X}_1), A(\mathcal{X}_2)] \leqslant d_\chi(\mathcal{X}_1, \mathcal{X}_2). \tag{17}$$

1.4.7 Joint Differential Privacy

Joint Differential Privacy (JDP) is proposed to prevent personal information that can be identified by other participants instead of an attacker. Given a patient p, JDP requires that the joint distribution on the anonymized results provided to other patients, *i.e.* $A(\mathbf{X})_p$, holds differential privacy on patient p's anonymized result.

$$\mathcal{L}_{\text{JDP}} = \forall \mathcal{S} \subseteq \text{Range}(A) : \Pr[A(\mathcal{X}_1)_p \in S \in \mathcal{X}] \leqslant \exp(\epsilon) \times \Pr[A(\mathcal{X}_2)_p \in S \in \mathbf{X}] + \delta. \tag{18}$$

1.5 Other Privacy Models for Relational Data

1.5.1 (X, Y)-Anonymity

(X, Y)-Anonymity (XYA) [160] enhances k-anonymity to limit the confidence with which values of sensitive attributes can be inferred. In this model, X and Y respectively refer to the groups of database columns with quasi-identifiers, and sensitive properties, $\mathbf{X}[x]$ denotes the number of records in the database $\mathbf{X}$ containing the value x. The percentage of records containing both value x and y with respect to those containing value x is required to be less than k.

$$\mathcal{L}_{\text{XYA}} = k, \quad \text{s.t. } y \in Y \left(x \in X \left(\frac{\mathbf{X}[y, x]}{\mathbf{X}[x]} \right) \right) \leqslant k \wedge k \in (0, 1]. \tag{19}$$

In a healthcare scenario, suppose we are given two releases of time-series medical data, then XYA would utilize common columns between two releases as X and thereby is able to make sure the two time-series releases satisfy (X, Y)-anonymity.

1.5.2 Normalized Variance

When data perturbation is adopted as a data anonymization method, Normalized Variance (NV) [161] can be employed to measure the dispersion between the raw data $\mathbf{X}$ and the perturbed data $\mathbf{X}^{'}$. The limitation of NV is that it does not account for the nature of the data itself, as it is designed based on the hypothesis that a high variance indicates a higher degree of privacy.

$$\mathcal{L}_{\text{NV}} = \frac{\sigma^2(\mathbf{X} - \mathbf{X}^{'})}{\sigma^2(\mathbf{X})}. \tag{20}$$

1.5.3 δ-disclosure privacy

δ-disclosure privacy (DDisP) is proposed to protect attribute disclosure by ensuring the distance between the distributions of sensitive values with a stricter definition compared to t-closeness. (*i.e.* multiplicative definition) [162]. DDisP can be defined as: an equivalent class $e \in \mathcal{E}$ holds δ-disclosure privacy with respect to the sensitive attribute s, if $\forall s \in S$:

$$\mathcal{L}_{\text{DDisP}} = \left| \log \frac{\Pr[e, s]}{\Pr[\mathbf{X}, s]} \right| < \delta. \tag{21}$$

And the data $\mathbf{X}$ holds the δ-disclosure privacy if for each $\mathcal{X} \in \mathcal{E}_Q$, then e is δ-disclosure private.

1.5.4 (d, γ)-privacy

During the process of data anonymization, privacy breaches may occur. The level of privacy breach can be measured if the posterior probability of a property, when the prior probability is available, is larger than a crisp valued threshold [163]. (d, γ)-privacy (DGP) [164] extends such a model by adding boundaries to the prior and posterior probabilities using d and γ respectively.

$$\mathcal{L}_{\text{DGP}} = \frac{d}{\gamma} \leqslant \frac{\Pr[s|S]}{\Pr[s]}, s.t. \Pr[s] \leqslant d, \Pr[s|S] \leq \gamma. \tag{22}$$

1.5.5 δ-presence

Essentially, this privacy model is used to protect membership disclosure [165]. Concretely, this model aims to bounds the attacker's probability of inferring the existence of an individual patient p in the anonymized and published data $\mathbf{X}^{'}$, based on the hypothesis that an external data $\mathbf{X}^E$ contains all the individual's information in $\mathbf{X}^{'}$. This model can be expressed as:

$$\mathcal{L}_{\text{DPR}} = (\underline{\delta}, \bar{\delta}), \quad \text{s.t. } \forall p \in \mathcal{P}^E : \underline{\delta} \leqslant \Pr[p \in \mathcal{P}^{'}] \leqslant \bar{\delta}, \tag{23}$$

where $\underline{\delta}$ and $\bar{\delta}$ represent the minimum and maximum values of δ, $\Pr(p \in \mathcal{P}^{'}) = \frac{\mathcal{P}^{'}}{\mathcal{P}^E}$ is commonly adopted by attackers to calculate the probability via the comparison of the number of users in two data. In the real world problem domain, this is not practical nor feasible [57]. For instance, if the anonymized data $\mathbf{X}^{'}$ is stored on the private servers of NHS trust, it is difficult for attackers to find the relevant data $\mathbf{X}^E$ for conducting such an inference.

1.5.6 Population uniqueness

Similar to the average risk model, this model is also threshold-based by ensuring the proportion of records that are unique within the underlying population [166]. To this end, basic information about the population is required, *e.g.* the sampling fraction and population size. Then, some statistical super-population models can be employed to estimate the characteristics of the entire population with probability distributions that are parameterized with sample characteristics. Typical models include Pitman [167], Zayatz [168], and SNB [169]. Practically, in the Pitman model, the sampling fractions is usually valued less than 10%. Given n data samples $\mathcal{X}_1, \mathcal{X}_2, \cdots, \mathcal{X}_n$ drawn from $\mathbf{X}$, the population uniqueness (PU) is expressed as the percentage of unique data samples. This is usually achieved by either the mean of uniqueness of an individual data record, or down-sampling a synthetic population of n individual records in line with the copula distribution. The former can be written as:

$$\begin{aligned}\mathcal{L}_{\text{PU}} &= \frac{1}{n}\mathbb{E}\left[\sum_{i=1}^{n}\left[\mathcal{X}_i \text{ is unique in } (\mathcal{X}_1, \mathcal{X}_2, \cdots, \mathcal{X}_n)\right]\right] \\ &= \frac{1}{n}\sum_{\mathcal{X}\in\mathbf{X}} \mathbb{E}[I_{\mathcal{X}}],\end{aligned} \tag{24}$$

where $I_{\mathcal{X}}$ is a Boolean indicator s.t. $I_{\mathcal{X}} = [\exists! i, \mathcal{X}_i = \mathcal{X}] = 1$ if there exists an individual record i holds $\mathcal{X}_i = \mathcal{X}$, and zero otherwise. Noteworthy, $I_{\mathcal{X}}$ usually follows a binomial distribution $B\Big(f(\mathcal{X}), n\Big)$. Thereby, Eq. (24) can be rewritten as

$$\mathcal{L}_{\text{PU}} = \sum_{\mathcal{X}\in\mathbf{X}} f(\mathcal{X})\Big[1 - f(\mathcal{X})\Big]^{n-1}. \tag{25}$$

However, this method is computationally expensive due to the iterative operations over all the combinations of data attributes in $\mathbf{X}$. Thus the former method is frequently employed in practice due to its better computational efficiency, which can be calculated as

$$\mathcal{L}_{\text{PU}} = \frac{1}{n}\left|\Big\{ i \in [1, n] / \forall i \neq j, \mathcal{X}_i \neq \mathcal{X}_j \Big\}\right|, \tag{26}$$

where n data records are drawn based on the cumulative marginal distributions and copula correlation matrix (which can be estimated via a randomized Quasi Monte Carlo approach [170]).

1.5.7 Sample uniqueness

The Sample Uniquess (SU) model is frequently used to restrict the fraction of records that are unique considering the indirect (*i.e.* quasi) identifiers. Similar to the population uniqueness introduced in Section 1.5.6, SU can be described as

$$\begin{aligned}\mathcal{L}_{\text{SU}} &= f_{\mathbf{X}}\Big[\mathcal{X} \text{ is unique in } (\mathcal{X}_1, \mathcal{X}_2, \cdots, \mathcal{X}_n)\Big]\Big| \exists i, \mathcal{X}_i = \mathcal{X} \\ &= f_{\mathbf{X}}[\forall i \in [2, n], \mathcal{X}_i \neq \mathcal{X}] \\ &= [1 - f(\mathcal{X})]^{n-1}.\end{aligned} \tag{27}$$

1.5.8 Profitability

This model implements a game-theoretic approach [171] for performing cost and benefit analysis of data sharing by maximising the monetary benefit of the data publisher.

2 Privacy Model for Transactional Data

In the real-world problem domain, the number of quasi-identifiers can be very large, which makes it difficult to adopt k-anonymity and guarantee the usefulness of the anonymized data. To cope with this issue in real-world transactional data with high dimensionality, k^m-Anonymity is designed in [172] by requiring each combination of up to m quasi-identifiers to appear at least k times in the anonymized data. The key idea of k^m-anonymity compared to k-anonymity is that there is limited privacy gain from protection against patients or adversary attackers who know most of the terms within a single record, and significant information loss in the effort for doing so. However, k^m-anonymity is unable to protect combinations with length larger than m. Additionally, the global generation of a priori anonymization may results in severe information loss.

3 Privacy Model for Relational-Transactional Data

In real-world medical problems, relational and transactional data is very common. To anonymize this type of data, (k, k^m)-anonymity is firstly proposed in [173] by ensuring that each equivalent class satisfies k-anonymity on relational data, while satisfying k^m-anonymity on transaction data. The drawback of (k, k^m)-Anonymity is three-fold: 1) it only protects combinations with length at most m in the transactional data; 2) it does not support diversity constraints and thus is not able to protect against homogeneous attacks [126]; 3) the requirements are too strict for equivalent classes, which results in severe information loss.

4 Privacy Model for Graph Data

A social network is usually represented by a graph in which nodes denoting individuals and edges denoting a relationship among individuals. In graph theory, a connected graph G is an ordered pair of vertices V and edges E, *i.e.* $G = (V, E)$. The overall objective of anonymized graph data is to achieve the adequate degree of privacy while minimising the number of vertices to be revised or deleted.

4.1 k-degree

In the context of social networks, link re-identification is a challenging issue for user privacy protection. It usually occurs when the sensitive information passing through the graph is accessed by unauthorized parties. Inspired by k-anonymity (in Section 1.1), k-degree anonymization [174] is proposed to prevent identity disclosure in social networks. This is achieved by removing the identity of a user via a social network graph. Thus, k-degree anonymization is essentially a graph anonymization algorithm in which the graph is k degree anonymous if there exists at least $k-1$ nodes with the same degree. In other words, all the vertices in the graph satisfy k-anonymity. However, typical friendship [175] and structure attacks [176] are still not well managed in k-degree anonymization. These two attacks belong to link disclosure [177] which may occur when attackers are able to determine any sensible relationship between social network users or a relationship that users expect to hide. In fact, in the problem domain of digital healthcare, this also exists as, for example, where the relationship between family members has been detected, some predictions required by attackers may make a reference to another family member.

4.2 k^2-degree

The friendship attack [175] is based on the node degree of all the nodes connected via an edge. The attacker on a social graph G utilizes the degree of the two endpoints of an edge to re-identify users and figures out their relationship based on the available information in the network. It is relatively easy for an attacker to identify users from candidate nodes when the network is small scale. To this end, k^2-degree anonymity [175] is proposed by requiring for every node with degree pair d, the existence of at least $k-1$ nodes with the same degree pair d in G. k^2-degree can also be treated as an optimization problem for small scale networks.

4.3 k-automorphism

Facing an anonymized social network, the target user or participant can still be recognized in the network by holding structural information, which leads to identity and link disclosure in structural attacks [178,179]. Practically, the attacker establishes a query in the given graph and this query matches with a limited number of nodes in the network, then the target person is uniquely identified in the network and this query is based on the structure of the target. To protect against such an attack, k-automorphism anonymity is devised by requiring there exist at least $k-1$ automorphic functions on the graph and for each node in the graph [176]. Using this anonymity, the attackers are unable to find node from the other $k-1$ symmetric nodes having any structural information with a probability higher than $1/k$, since there does not exist any structural differences among the nodes and the other $k-1$ symmetric ones.

4.4 (k, l)-anonymity

To minimise information loss and consider the re-identification risk that caused by an adversary, the first (k, l)-Anonymity (KLA) privacy model was defined in [180] based on the neighbourhood $N(\cdot)$ of the graph. Specifically, a graph G is $(k.l)$-anonymous if for each vertex $v \in V$, there exists a subset of vertices $U \subseteq V$ excluding v s.t. $|U| \geqslant k$ and for each element $u \in U$, the vertices u and v share at least l neighbours. This definition suffers from the risk of information disclosure and a lack of evaluation on real-world datasets. Thereby, [181] refined the original definition as: if for any vertex $v \in V$, and for any subset $S \subseteq N(v)$ of cardinality $|S| \leqslant l$, there exists at least k distinct vertices $\{v_i\}_{i=1}^{k}$ s.t. $S \subseteq N(v_i)$. In other words, a graph G satisfies KLA if for any subset S of at most l neighbours of a vertex v, there should exists a new vertex set of V with at least k vertices share common neighbours S.

$$\mathcal{L}_{\text{KLA}} = (k, l), \quad \text{s.t. } \forall v : v_i \in V, \forall S : S \subseteq N(v_i), |S| \leqslant l, i \in [1, k]. \tag{28}$$

The above formulation employed in [181] requires a random clone process of problematic vertices until G meets the criteria of KLA. That is, the algorithm is required to insert several new vertices with the same neighbourhood of the problematic vertices. This simple solution could result in the algorithm to lose its privacy. As a consequence, from the adversary perspective, the re-identification probability could be increased higher than $1/k$ if the anonymization algorithm and the neighbourhood of vertices are known. Followed by those observations, enhanced methods have been proposed in the literature. In particular, a practical KLA is implemented in [67] by adding a large number of edges to the graph for guaranteeing the privacy level and then to tailor the redundant ones to minimising information loss. Therefore, it is a good practice to leverage the re-identification risk and degree of usability of the anonymized graph.

5 Privacy Model for Geolocational Data

The Historical k-Anonymity (HKA) privacy model [182] is proposed to meet the increased demand of GPS services by defining $\langle time, location \rangle$ pairs as quasi-identifiers and block the link request to attackers except k or more end users. In this model, the personal history of geolocations L is essentially a sequence of $\langle time, geolocation \rangle$ pairs, and requests r are times and geolocations which the device of the end patient (or user) requested. L is time-geolocation consistent with r in the case when there exists an entry in L whose time and location are within the time interval and geographical area provided in R. HKA can be satisfied if the set of patient requests $\mathcal{R}_p$ is location-time consistent with the location history of $k-1$ other patients $\mathcal{P}$.

$$\mathcal{L}_{\text{HKA}} = k, \quad \text{s.t. } \forall p, p^{'} \in \mathcal{P} : |L_{p'} \text{is geolocation-time consistent with } |R_p| \geqslant k. \tag{29}$$
