# Supplementary material for: Data Anonymization for Pervasive Health Care: Systematic Literature Mapping Study
Source: JMIR Med Inform. 2021 Oct 15;9(10):e29871. doi: 10.2196/29871 (PMC8556642; doi:10.2196/29871)
Supplement: Multimedia Appendix 5 [file medinform_v9i10e29871_app5.pdf]

Multimedia Appendix 5

Usability metrics for privacy models

1 Usability Metrics for Numerical and Categorical Data

1.1 Information Loss and Its Variants

Information Loss (IL) [223] is frequently employed for measuring the amount of distorted or eliminated information in the anonymized data $\mathbf{X}^{'}$, in comparison to the raw data $\mathbf{X}$. In other words, information loss is used to quantify the degree of privacy gain that would occur (with respect to the original data set) should the information be disclosed. The overall IL is essentially taken in the form of the weighted summation of both the loss of the numerical attributes and categorical attributes. Suppose we are given a set of quasi-identifiers (*i.e.* attributes) $Q = \{q_1, \cdots, q_d\}$ and an equivalent class $\dot{\mathcal{E}} \in \mathcal{E}$, a numerical attribute $N \in Q$, let $[U_N, V_N]$ and $[u_N^{\dot{\mathcal{E}}}, v_N^{\dot{\mathcal{E}}}]$ be the corresponding domain and value range in $\dot{\mathcal{E}}$. For a categorical attribute C that relies on a pre-defined hierarchy $\dot{H}$, suppose that $\underline{a}$ is the lowest ancestor of all the Cs in $\dot{\mathcal{E}}$, $\xi(\underline{a})$ and $\xi(\dot{\mathcal{H}}_C)$ represents the set of leaves under $\underline{a}$ and set of all the leaves under the $\dot{\mathcal{H}}_C$. To this end, the IL regarding the N and C in $\dot{\mathcal{E}}$ can be expressed as:

$$\begin{cases} \mathcal{U}_{\text{IL}}^{\text{N}}(\dot{\mathcal{E}}) = \frac{v_N^{\dot{\mathcal{E}}} - u_N^{\dot{\mathcal{E}}}}{V_N - U_N} & \text{Numerical Attribute,} \\ \mathcal{U}_{\text{IL}}^{\text{C}}(\dot{\mathcal{E}}) = \frac{|\xi(\underline{a})|}{|\xi(\dot{\mathcal{H}}_C)|} & \text{Categorical Attribute.} \end{cases} \tag{1}$$

It is noticeable that $\mathcal{U}_{\text{IL}}^{\text{C}}(\dot{\mathcal{E}}) = 0$ *iff* $|\xi(\underline{a})| = 1$. Thus, the overall information loss regarding the $\dot{\mathcal{E}}$ can be defined as:

$$\mathcal{U}_{\text{IL}}(\dot{\mathcal{E}}) = \sum_{i=1}^{d} w_i \times \mathcal{U}_{\text{IL}}^{q_i}(\dot{\mathcal{E}}), \tag{2}$$

where w_i is the weight associated with q_i, s.t. $\sum_{i=1}^{d} w_i = 1$, and w_i is usually valued as $1/d$ in practice. Then, we are able to further calculate the Average Information Loss (AIL) on $\mathbf{X}$, which anonymized as $\mathbf{X}^{'}$, a set of equivalent classes $\mathcal{E}$:

$$\mathcal{U}_{\text{AIL}}(\mathcal{E}) = \frac{\sum_{\dot{\mathcal{E}} \in \mathcal{E}} |\dot{\mathcal{E}}| \times \mathcal{U}_{\text{IL}}(\dot{\mathcal{E}})}{|\mathbf{X}|}. \tag{3}$$

Similarly, the maximum information loss (MIL) can be calculated by generalizing each attribute q_i to the entire domain $\mathcal{D}_i$:

$$\mathcal{U}_{\text{MIL}}(\mathcal{E}) = \sum_{i=1}^{d} -\log\left(\frac{1}{\mathcal{D}_i}\right). \tag{4}$$

Generalized Information Loss (GIL) [224] is a metric used to capture the penalty incurred during the process of generalizing a specific attribute, by quantifying the fraction of the domain values that have been generalized [224]. The underlying idea is that a larger range of values (described by data values) are less

precise compared to smaller range ones, however, the data structure cannot be evaluated. In practice, its normalized version [225] is more commonly adopted. Suppose we are given the original data $\mathbf{X} \in \mathbb{R}^{m \times n}$, then the GIL regarding the anonymized data $\mathbf{X}^{'}$ is defined as follows:

$$\mathcal{U}_{\mathrm{GIL}}\left(\mathbf{X}^{'}\right) = \frac{1}{m \cdot n} \sum_{i=1}^{n} \sum_{j=1}^{m} \frac{V_{ij} - U_{ij}}{V_i - U_i}, \tag{5}$$

where, similar to IL, the V_i and U_i are corresponding to the upper and lower bounds of the attribution q_i, ij refers to the interval of upper and lower bound ending points. Concretely, $\mathcal{U}_{\mathrm{GIL}}(\mathbf{X}^{'}) = 0$ and $\mathcal{U}_{\mathrm{GIL}}(\mathbf{X}^{'}) = 1$ indicating the no anonymization (or transformation) and full suppression (or generalization) on the raw data $\mathbf{X}$. This metric has been confirmed to be suitable for both hierarchical and non-hierarchical algorithms [125] such as Mondrian.

1.2 Privacy Gain

Privacy gain (PG) is commonly used to minimise information loss while maximising privacy gain in the practical data anonymization process. Given the raw data $\mathbf{X}$ and the anonymized data $\mathbf{X}^{'}$:

$$\mathcal{U}_{\mathrm{PG}}\left(\mathbf{X}^{'}\right) = \mathrm{avg}\left\{\mathcal{L}(\mathbf{X}) - \mathcal{L}(\mathbf{X}^{'})\right\}, \tag{6}$$

where $\mathcal{L}(\cdot)$ denotes the level of anonymity. Concretely, each quasi-identifier $q_i \in Q$ will be used in the calculation. In addition, the information loss and privacy gain can be combined for leveraging both sides, *i.e.* ILPG [226], which can be taken in the form of:

$$\mathcal{U}_{\mathrm{ILPG}}\left(\mathbf{X}^{'}\right) = \frac{\mathcal{U}_{\mathrm{IL}}(\dot{\mathcal{E}})}{\mathcal{U}_{\mathrm{PG}}(\dot{\mathcal{E}})}. \tag{7}$$

1.3 Discernibility

Discernibility Metric (DM) is a metric used to measure how indistinguishable a record is from others, by assigning a penalty to each record, equal to the size of the equivalence class to which it belongs [227]. If a record is suppressed, then it is assigned a penalty equal to the size of the input table. Suppose we are given the original data $\mathbf{X} \in \mathbb{R}^{m \times n}$, then the DM regarding the anonymized data $\mathbf{X}^{'}$ is computed as:

$$\mathcal{U}_{\mathrm{DM}}\left(\mathbf{X}^{'}\right) = \sum_{\forall \mathcal{E} \text{ s.t. } \left|\dot{\mathcal{E}}\right| \geq k} \left|\dot{\mathcal{E}}\right| + \sum_{\forall \mathcal{E} \text{ s.t. } \left|\dot{\mathcal{E}}\right| < k} m \cdot \left|\dot{\mathcal{E}}\right|, \tag{8}$$

where $|\dot{\mathcal{E}}|$ represents the size of the equivalent class, *i.e.* the anonymized groups in $\mathbf{X}^{'}$. The lower value of the $\mathcal{U}_{\mathrm{DM}}(\mathbf{X}^{'})$, the better privacy guaranteed. This is due to the fact that larger $\mathcal{E}$ indicates more information loss.

1.4 Average Equivalence Class Size

Average Equivalence Class Size Metric (AECSM) is a metric designed to reflect how well the creation of the equivalence class $\mathcal{E}$ approaches the best anonymization result, where each record is generalized in an $\dot{\mathcal{E}}$ of k records [124]. Given original data $\mathbf{X} \in \mathbb{R}^{m \times n}$, the AECS is defined as:

$$\mathcal{U}_{\mathrm{AECSM}}\left(\mathbf{X}^{'}\right) = \frac{m}{|\mathcal{E}| \cdot k}, \tag{9}$$

where m is the number of data records in the anonymized data $\mathbf{X}^{'}$, $|\mathcal{E}|$ denotes the total number of equivalent classes. This metric does not depend on the generalization hierarchies.

1.5 Matrix Norm

Matrix Norm (MN) [228] is commonly adopted to measure how much a non-zero vector can be stretched by a matrix in linear algebra, expressed as:

$$\begin{aligned}\mathcal{U}_{\mathrm{MN}}\left(\mathbf{X},\mathbf{X}^{'}\right) &= \sum_{i=1}^{m}\sum_{j=1}^{n}\left|\tilde{x}_{ij} - \tilde{x^{'}}_{ij}\right| \\ &= \sum_{i=1}^{m}\sum_{j=1}^{n}\frac{\left|x_{ij} - x^{'}_{ij}\right|}{\sigma_j},\end{aligned} \tag{10}$$

where matrix element $x_{ij} \in \mathbf{X}$ and $x^{'}_{ij} \in \mathbf{X}^{'}$, $\sigma_j = \sqrt{\frac{\sum_{i=1}^{m}(x_{ij}-\mu_j)^2}{m}}$ denotes the standard deviation in which the mean $\mu_j = \frac{1}{m}\sum_{i=1}^{m} x_{ij}, i \in [1, m], j \in [1, m]$. Alternatively, Eq. (10) can also be written in the $\ell 1$ or $\ell 2$ norms [229]. In general, the smaller of the $\mathcal{U}_{\mathrm{MN}}(\mathbf{X}, \mathbf{X}^{'})$, the higher degree of usefulness of the anonymized data $\mathbf{X}^{'}$. On the contrary, larger $\mathcal{U}_{\mathrm{MN}}(\mathbf{X}, \mathbf{X}^{'})$ connotes the matrix itself is more *ill-conditioned.*

1.6 Correlation

Correlation Metric (CM) [230] is usually defined to measure the dependence between two pairs of vectors in probability theory, and thereby, can be treated as similarity metric. Note that the correlation analysis can only be applied on vectors, thus original data $\mathbf{X}$ and anonymized data $\mathbf{X}^{'}$ should be converted to vector forms $\overrightarrow{\cdot}$ respectively first and then the CM could be computed as follows:

$$\begin{aligned}\mathcal{U}_{\mathrm{CM}}\left(\mathbf{X},\mathbf{X}^{'}\right) &= \mathcal{U}_{\mathrm{CM}}\left(\overrightarrow{\mathbf{X}},\overrightarrow{\mathbf{X}^{'}}\right) \\ &= \frac{\sum_{i=1}^{mn}\left(\overrightarrow{x_i} - \mu_{\overrightarrow{\mathbf{X}}}\right)\left(\overrightarrow{x^{'}_i} - \mu_{\overrightarrow{\mathbf{X}^{'}}}\right)}{\sqrt{\sum_{i=1}^{mn}\left(\overrightarrow{x_i} - \mu_{\overrightarrow{\mathbf{X}}}\right)^2\sum_{i=1}^{mn}\left(\overrightarrow{x^{'}_i} - \mu_{\overrightarrow{\mathbf{X}^{'}}}\right)^2}},\end{aligned} \tag{11}$$

where $\overrightarrow{x_i} \in \overrightarrow{\mathbf{X}}$ and $\overrightarrow{x^{'}_i} \in \overrightarrow{\mathbf{X}^{'}}$, $\mu_{\overrightarrow{\mathbf{X}}}$ and $\mu_{\overrightarrow{\mathbf{X}^{'}}}$ are the mean of the vector $\overrightarrow{\mathbf{X}}$ and $\overrightarrow{\mathbf{X}^{'}}$. In general, the larger of the $\mathcal{U}_{\mathrm{CM}}(\mathbf{X}, \mathbf{X}^{'})$, the higher degree of usefulness of the anonymized data $\mathbf{X}^{'}$.

1.7 Divergence

Kullback–Leibler Divergence (KLD) [231] is a common metric used to measure the distance between two probability distributions. In probability theory, the observed data with a complex distribution is often replaced by an approximated one. On this basis, KLD helps to quantify information loss during the selection of such an approximation [232], which can be defined as:

$$\begin{aligned}\mathcal{U}_{\mathrm{KLD}}\left(\mathbf{X},\mathbf{X}^{'}\right) &= \mathcal{U}_{\mathrm{KLD}}\left(\Phi(x)\middle\|\Theta(x^{'})\right) \\ &= \int_x \Phi(z)\log\frac{\Phi(z)}{\Theta(z)}dz \\ &= \frac{1}{2}\left(\mathrm{tr}(\Xi_x\Xi_{x^{'}}^{-1}) - \log\frac{\det(\Xi_x)}{\det(\Xi_{x^{'}})} - n + (\mu_x - \mu_{x^{'}})^\top\Xi_{x^{'}}^{-1}(\mu_x - \mu_{x^{'}})\right),\end{aligned} \tag{12}$$

where $\Phi(x)$ and $\Theta(x^{'})$ represent the distribution of two multivariate random variables $x \in \mathbf{X}$ and $x^{'} \in \mathbf{X}^{'}$, $\mathrm{tr}(\cdot)$ and $\det(\cdot)$ respectively denotes the trace and determinant of a matrix, μ_x and $\mu_{x'}$ are the mean. Note that, Ξ_x and $\Xi_{x'}$ are the autocovariance matrices that could be estimated. In general, the larger of the $\mathcal{U}_{\mathrm{KLD}}(\mathbf{X}, \mathbf{X}^{'})$, the lower degree of usability of the anonymized data $\mathbf{X}^{'}$ due to greater information loss.

2 Usability Metrics for Image Data

It is observed from the work [62] that a dataset is usually organized in 2-D table, and thus can be expressed as a gray-scale image. To this end, we are inspired to revisit several image quality assessment metrics that originally proposed in the researches of signal processing and image processing. There are two categories of image quality assessment methods: subjective and objective. The former category is based on the judgement of human visual perception and the latter is proposed from the machine vision perspective. In this work, we focus on the objective ones and for readers interested in subjective one please refer to [A5_1,A5_2,243] for details. In particular, PSNR (Section 2.2) and SSIM (Section 2.3) are the two most commonly metrics are still in use at present within the realm of computer vision [234,242,244,A5_3,A5_4,A5_5].

2.1 Mean Squared Error and Mean Absolute Error

Mean Squared Error (MSE) is a $\ell 2$ norm (*i.e.* $\|\cdot\|_2^2$) that usually employed to describe the differences between a pair of images – the raw and anonymized data in the problem domain of data anonymization. Contrastly, Mean Absolute Error (MAE) is taken in the $\ell 1$ norm (*i.e.* $\|\cdot\|$).

$$\mathcal{U}_{\mathrm{MSE}}\Big(\mathbf{X}, \mathbf{X}^{'}\Big) = \frac{1}{mn} \sum_{i=1}^{m} \sum_{j=1}^{n} \Big(x_{ij} - x_{ij}^{'}\Big)^2. \tag{13}$$

Obviously, the smaller of the MSE and MAE, the higher degree of usability of the anonymized data $\mathbf{x}^{'}$. In practice, MAE (*e.g.* [235] and [236]) and MSE (*e.g.* [233] and [234]) are commonly used as part or whole of the loss function in training end-to-end learning models for improving its robustness and generality.

2.2 Peak Signal-to-Noise Ratio

Peak Signal-to-Noise Ratio (PSNR) [237,238] is frequently employed as a image quality measurement between the original image and a compressed or reconstructed image. In this work, we use original and anonymized data taken in the form of gray-scale image accordingly and define PSNR as:

$$\mathcal{U}_{\mathrm{PSNR}}\Big(\mathbf{X}, \mathbf{X}^{'}\Big) = 10\log_{10} \frac{\phi^2}{\mathcal{U}_{\mathrm{MSE}}(\mathbf{X}, \mathbf{X}')}, \tag{14}$$

where ϕ is the maximum pixel value and valued as either 255 (resolution of 8 bit per pixel) or 1 (each pixel value is within the range of $[0, 1]$). Note that PSNR is unbounded, *i.e.* $\mathcal{U}_{\mathrm{PSNR}} \in [0, +\infty)$. Concretely, $\mathcal{U}_{\mathrm{PSNR}}(\mathbf{X}, \mathbf{X}) = +\infty$ *iff* $\mathcal{U}_{\mathrm{MSE}}(\cdot) = 0$, thus we suggest to set 100 as the MSE calculation result in this case. Additionally, both MSE and PSNR are robust quantitative measurements yet are inconsistent with human visual perception [239,240,241]. In addition, PSNR is also considered in training deep neural networks by using $\ell 1$ loss function [242].

2.3 Structural Similarity Index

Structural Similarity Index (SSIM) [243], which differs from the conventional error summation methods (*e.g.* MAE, MSE, etc), is proposed to model image distortion as a combination of three factors (*i.e.* luminance distortion L, contrast distortion C, and loss of correlation S) and can be computed as:

$$
\begin{aligned}
\mathcal{U}_{\text{SSIM}}\left(\mathbf{X},\mathbf{X}^{'}\right) &= L\left(\mathbf{X},\mathbf{X}^{'}\right)C\left(\mathbf{X},\mathbf{X}^{'}\right)S\left(\mathbf{X},\mathbf{X}^{'}\right) \\
&= \frac{2\mu_{\mathbf{X}}\mu_{\mathbf{X}'}+C_1}{\mu_{\mathbf{X}}^2+\mu_{\mathbf{X}'}^2+C1}\times\frac{2\sigma_{\mathbf{X}}\sigma_{\mathbf{X}'}+C_2}{\sigma_{\mathbf{X}}^2+\sigma_{\mathbf{X}'}^2+C2}\times\frac{\sigma_{\mathbf{X}\mathbf{X}'}+C_3}{\sigma_{\mathbf{X}}\sigma_{\mathbf{X}'}+C3},
\end{aligned}
\tag{15}
$$

where $\mu_{\mathbf{X}}$ and $\mu_{\mathbf{X}'}$ are the mean value of luminance of $\mathbf{X}$ and $\mathbf{X}^{'}$, $\sigma_{\mathbf{X}}$ and $\sigma_{\mathbf{X}'}$ are the standard deviations, $\sigma_{\mathbf{X}}^2$ and $\sigma_{\mathbf{X}'}^2$ are the variance, $\sigma_{\mathbf{X}\mathbf{X}'}$ denotes the covariance between $\mathbf{X}$ and $\mathbf{X}^{'}$, $C_1, C2$, and C_3 are the positive constants added to avoid the situation when the denominator is very close to zero [244]. Different to PSNR, SSIM is bounded, *i.e.* $\mathcal{U}_{\text{SSIM}}(\mathbf{X},\mathbf{X}^{'}) \in [0,1]$ and its optimal parameter values can be found in [243]. The higher of the SSIM calculation result, the higher utility of the anonymized data $\mathbf{X}^{'}$. For readers who are interested in adopting SSIM in training deep neural networks, the Eq. 15) is recommended by [244] as the SSIM loss function for performance enhancement.
