# Supplementary material for: Data Anonymization for Pervasive Health Care: Systematic Literature Mapping Study
Source: JMIR Med Inform. 2021 Oct 15;9(10):e29871. doi: 10.2196/29871 (PMC8556642; doi:10.2196/29871)
Supplement: Multimedia Appendix 6 [file medinform_v9i10e29871_app6.pdf]

Multimedia Appendix 6

Re-identification risk metrics for privacy models

1 Average risk, Overall Risk and β-Likeness

Average risk is a constraint of re-identifying the records using a crisp valued threshold. It is the most fundamental risk assessment used in both theoretical and practical data anonymization. The accumulated average risk is the overall risk. β-Likeness has been introduced in Multimedia Appendix 4.

2 Distance Linked Disclosure and Related Measurements

Distance Linked Disclosure (DLD) [219] is defined to measure the risk of privacy loss. Specifically, DLD is the average percentage of linked data records using distance-based record linkage where the average percentage is calculated over a number of important variables that the attackers are assumed to know. This is usually achieved by measuring the linkage between the original record and the closest protected record using a distance metric such as Euclidean distance. Suppose we are given the raw data $\mathbf{X}$ and the anonymized data $\mathbf{X}^{'}$, and let $|\langle\mathbf{X}\cap\mathbf{X}^{'}\rangle|$ be the number of records which can be linked in $\mathbf{X}^{'}\in\mathbb{R}^{m\times n}$, thus:

$$\mathcal{R}_{\text{DLD}}=\frac{|\langle\mathbf{X}\cap\mathbf{X}^{'}\rangle|}{m}. \tag{1}$$

Similar to DLD, there is Probabilistic Linkage Disclosure (PLD) [220] risk, which also calculates the average percentage, but using probabilistic-based record linkage. Another measurement is called Interval Disclosure (ID) [221], which is the average percentage of original values in the raw data $\mathbf{X}$ that are valued within the interval around the corresponding anonymized values in $\mathbf{X}^{'}$. These three measurements can work jointly with Information Loss for trading-off the risk and privacy loss.

3 Log-linear models

Log-linear Models (LLM) [222] are a global risk measurement that is based on the hypothesis that sample frequencies are independent and follow a Poisson distribution in which the corresponding parameters are estimated via the log-linear model. LLM aims to figure out the number of samples that are unique within the population with a Poisson distribution model, and estimate the number of correct matches for sample uniques (*i.e.* records that are unique within the sample). Concretely, suppose sample frequencies, by considering O patterns, can be easily modelled using a Poisson distribution. To this end, a global risk $\mathcal{R}_{\text{LLM}}$ can be defined as:

$$\mathcal{R}_{\text{LLM}}=\sum_{o=1}^{O}\exp\Big(-\frac{\gamma_o(1-\nu_o)}{\nu_o}\Big),\ \text{s.t.}\ \gamma_m=\nu_o\lambda_o, \tag{2}$$

where γ_o and its associated regression coefficient can be estimated by the LLM via iterative proportional fitting[1].

[1]https://cran.r-project.org/web/packages/sdcMicro/vignettes/sdc_guidelines.pdf
