# Supplementary material for: Data Anonymization for Pervasive Health Care: Systematic Literature Mapping Study
Source: JMIR Med Inform. 2021 Oct 15;9(10):e29871. doi: 10.2196/29871 (PMC8556642; doi:10.2196/29871)
Supplement: Multimedia Appendix 7 [file medinform_v9i10e29871_app7.pdf]

Multimedia Appendix 7

Ablation study for privacy-usability trade-offs and practical feasibility

In this multimedia appendix, we conduct an ablation study to further discuss *Privacy-Usability Trade-Offs and Practical Feasibility*, for Discussion 1. Specifically, We use two publicly available Electronic Health Records (EHR) datasets (briefed in Table 1) for classification tasks: **Breast Tissue Data Set** [297] (BTDS) and **Diabetic Retinopathy Debrecen Data Set** [298] (DRDDS). BTDS contains 106 data instances with 9 feature dimensions that can be classified into 6 categories including carcinoma, fibro-adenoma, mastopathy, glandular, connective, and adipose. DRDDS includes 1,151 data instances categorized into 2 classes: having Diabetic Retinopathy (DR) and not having DR.

Table 1: Summary of the two clinical data sets. † denotes the exclusion of class label.

Data Set	# of instances	# of dim.†	# of classes	Missing values?	Year published
BTDS [297]	106	9	6		2010
DRDDS [298]	1,151	19	2		2014

In this ablation study, we use the raw data from each of two datasets, along with two versions of anonymized data: dimensionality reduced by PCA and conventional privacy models. Concretely, we retain 7 out 9 attributes on BTDS and 15 out of 19 attributes on DRDDS. To obtain anonymized data from conventional privacy models, we use the tool ARX [135] to configure k-anonymity ($k = 1$) with l-diversity ($l = 1$) and (ϵ, δ)-differential privacy ($\epsilon = 1, \delta - 0.79$), and assign two ("Area" and "Max IP") and four ("0", "12", "13", and "15") Quasi-Identifying attributes respectively in BTDS and DRDDS. The remaining features are classed as sensitive attributes. After data anonymization, we remove these two columns resulting in 7 and 15 attributes respectively.

To keep consistency, we adopted PCA to reduce the dimension from 9 to 7 in BTDS and from 19 to 15 in DRDDS. For classifying the raw and anonymized datasets, three classifiers [299,300] are used, namely Back-Propagation Neural Network (BPNN), Gaussian Naïve Bayes (GNB) and Random Forest (RF), with performance measured using 10-Fold cross-validation. The results of classification are visualized in Fig. 1 and its privacy statistics are summarized in Table 2.

Table 2: A summary of re-identification risk and usability. Measured in %.

Data Set	Raw		PCA		Anon	
	ReID risk	Utility	ReID risk	Utility	ReID risk	Utility
BTDS [297]	91.51	100.00	0.00	N/A	50.00	99.61
DRDD [298]	69.85	100.00	0.00	N/A	4.82	99.73

Based on the above experimental results, we observe that data anonymized by the conventional privacy model (*i.e.* Anon) significantly outperforms the one anonymized by PCA and the raw data on the BTDS. However, the risk of re-identification of the PCA data set is much lower than Anon. In contrast, in DRDDS, the re-identification risk of Anon approaches PCA, however, the latter yields better classification

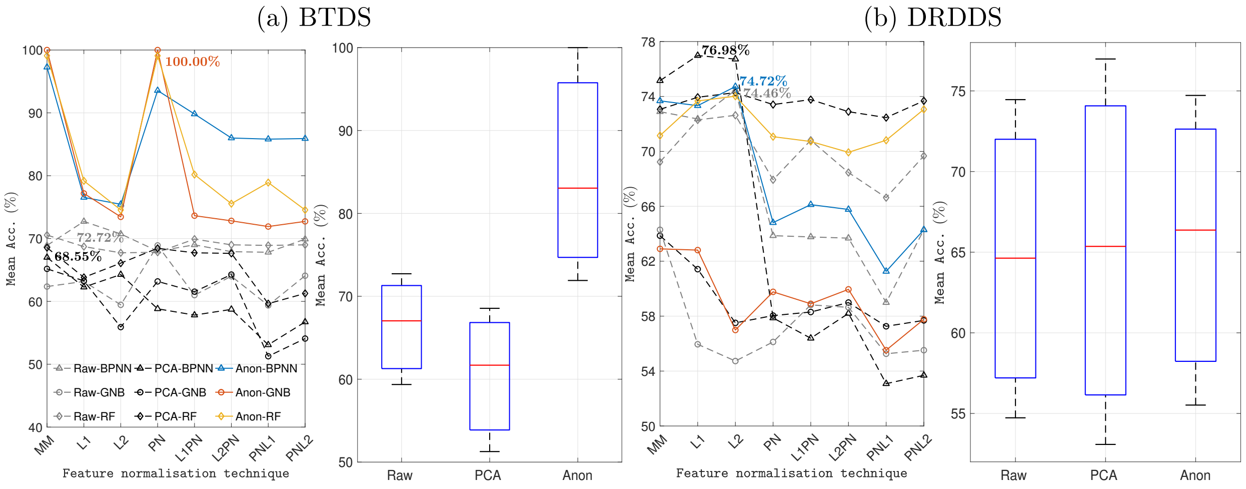

Figure 1: Classification accuracies of different feature selection methods in comparison to the raw data by varying both feature normalization methods and classifiers. For each data set, the left column shows the detailed classification performance, and the right column further summarizes the corresponding statistics. Best viewed in color.

performance.
